# Diagnosis and Treatment of Keloids and Hypertrophic Scars—Japan Scar Workshop Consensus Document 2018

**DOI:** 10.1186/s41038-019-0175-y

**Published:** 2019-12-27

**Authors:** Rei Ogawa, Sadanori Akita, Satoshi Akaishi, Noriko Aramaki-Hattori, Teruyuki Dohi, Toshihiko Hayashi, Kazuo Kishi, Taro Kono, Hajime Matsumura, Gan Muneuchi, Naoki Murao, Munetomo Nagao, Keisuke Okabe, Fumiaki Shimizu, Mamiko Tosa, Yasuyoshi Tosa, Satoko Yamawaki, Shinichi Ansai, Norihisa Inazu, Toshiko Kamo, Reiko Kazki, Shigehiko Kuribayashi

**Affiliations:** 1 Department of Plastic, Reconstructive and Aesthetic Surgery, Nippon Medical School, 1-1-5 Sendagi Bunkyo-ku, Tokyo, 113-8603 Japan; 2 Department of Plastic Surgery, Wound Repair and Regeneration, Fukuoka University, Fukuoka, 814-0180 Japan; 3 Department of Plastic Surgery, Nippon Medical School Musashikosugi Hospital, 1-396 Kosugicho, Nakahara-ku, Kawasaki-shi, Kanagawa 211-8533 Japan; 4 Department of Plastic and Reconstructive Surgery, Keio University School of Medicine, 35 Shinanomachi, Shinjuku-ku, Tokyo, 160-8582 Japan; 5 Department of Plastic and Reconstructive Surgery, Faculty of Medicine and Graduate School of Medicine, Hokkaido University, Kita-15, Nishi-7, Kita-ku, Sapporo, 060-8638 Japan; 6 Department of Plastic Surgery, Tokai University School of Medicine, 4-1-1 Kitakaname, Hiratsuka, Kanagawa 259-1292 Japan; 7 Department of Plastic and Reconstructive Surgery, Tokyo Medical University, 6-7-1 Nishishinjuku, Shinjuku-ku, Tokyo, 160-0023 Japan; 8 Department of Plastic and Reconstructive Surgery, Osaka Saiseikai Nakatsu Hospital, 2-10-39 Shibata, Kita-ku, Osaka, 530-0012 Japan; 9 Department of Plastic, Reconstructive and Aesthetic Surgery, Iwate Medical University School of Medicine, 19-1 Uchimaru, Morioka, Iwate 020-8505 Japan; 10 Department of Plastic Surgery, Oita University Hospital, 1-1 Idaigaoka, Hasamamachi, Yufu-shi, Oita 879-5503 Japan; 11 Department of Plastic Surgery, Showa University Fujigaoka Hospital, 1-30 Fujigaoka, Aoba-ku, Yokohama, Kanagawa Japan; 12 Department of Plastic and Reconstructive Surgery, Japanese Red Cross Fukui Hospital, 2-4-1 Tsukimi, Fukui, 918-8501 Japan; 13 Division of Dermatology and Dermatopathology, Nippon Medical School Musashikosugi Hospital, 1-396, Kosugicho, Nakahara-ku, Kawasaki-shi, Kanagawa 211-8533 Japan; 14 Faculty of Pharmaceutical Sciences, Teikyo Heisei University, 4-21-2 Nakano, Nakano-ku, Tokyo, 164-8530 Japan; 15 Wakamatsu-cho Mental and Skin Clinic, 9-4 Wakamatsu-cho, Shinjyuku-ku, Tokyo, 162-0056 Japan; 16 Department of Radiation Oncology, Nippon Medical School Hospital, Tokyo, 113-8603 Japan

**Keywords:** Keloid, Hypertrophic scars, Pathological scars, Guideline, Pathology, Surgery, Radiotherapy, Steroid, Laser

## Abstract

There has been a long-standing need for guidelines on the diagnosis and treatment of keloids and hypertrophic scars that are based on an understanding of the pathomechanisms that underlie these skin fibrotic diseases. This is particularly true for clinicians who deal with Asian and African patients because these ethnicities are highly prone to these diseases. By contrast, Caucasians are less likely to develop keloids and hypertrophic scars, and if they do, the scars tend not to be severe. This ethnic disparity also means that countries vary in terms of their differential diagnostic algorithms. The lack of clear treatment guidelines also means that primary care physicians are currently applying a hotchpotch of treatments, with uneven outcomes. To overcome these issues, the Japan Scar Workshop (JSW) has created a tool that allows clinicians to objectively diagnose and distinguish between keloids, hypertrophic scars, and mature scars. This tool is called the JSW Scar Scale (JSS) and it involves scoring the risk factors of the individual patients and the affected areas. The tool is simple and easy to use. As a result, even physicians who are not accustomed to keloids and hypertrophic scars can easily diagnose them and judge their severity. The JSW has also established a committee that, in cooperation with outside experts in various fields, has prepared a Consensus Document on keloid and hypertrophic scar treatment guidelines. These guidelines are simple and will allow even inexperienced clinicians to choose the most appropriate treatment strategy. The Consensus Document is provided in this article. It describes (1) the diagnostic algorithm for pathological scars and how to differentiate them from clinically similar benign and malignant tumors, (2) the general treatment algorithms for keloids and hypertrophic scars at different medical facilities, (3) the rationale behind each treatment for keloids and hypertrophic scars, and (4) the body site-specific treatment protocols for these scars. We believe that this Consensus Document will be helpful for physicians from all over the world who treat keloids and hypertrophic scars.

## Background

There has been a long-standing need for guidelines on the diagnosis and treatment of keloids and hypertrophic scars that are based on an understanding of the underlying disease mechanisms. The development of such guidelines has been greatly hampered by our poor understanding of the general pathomechanisms that drive these fibrotic scars and the molecular biological differences between keloids and hypertrophic scars. This is largely due to the difficulty in creating suitable animal models. Ethnic differences in pathological scarring propensity have also hampered the evolution of clear and globally useful diagnostic guidelines: Caucasians are much less prone to keloids and hypertrophic scars than Asians and Africans and if they do develop such scars, they tend not to be as drastic as those in more susceptible populations. These diagnostic problems have in turn severely obstructed the development of effective treatment algorithms. These issues have led each primary care physicians to diagnose and treat keloids and hypertrophic scars on the basis of their own perspective and experience, with the result that the current treatment outcomes are very uneven and occasionally deleterious to the patient.

To overcome this chaotic situation, the Japan Scar Workshop (JSW) has created a tool for objectively diagnosing keloids and hypertrophic scars. This tool is called the JSW Scar Scale (JSS) and it involves scoring the risk factors of individual patients and the affected areas. It is simple and easy to use and thus even physicians who are not accustomed to these pathological scars can easily diagnose them and judge their severity. The JSS 2011 version was announced in 2011, and the revised version JSS 2015 was announced in 2015.

Furthermore, the JSW has established a committee that, in cooperation with outside experts in various fields, has prepared a Consensus Document on keloid and hypertrophic scar treatment guidelines. This Consensus Document is contained in this article and is based on the currently available scientific literature and the experience of the contributing experts. It should be noted that the clinical evidence for many of the treatment guidelines is relatively sparse at present; consequently, the guidelines are likely to change over time as research and clinical experience progresses. The treatment guidelines in the present Consensus Document are easy to understand and will help even inexperienced clinicians to choose the most suitable treatment. It is thus likely to be useful for physicians from all over the world who treat keloids and hypertrophic scars.

The first part of this Consensus Document is I. Diagnostic algorithm for pathological scars and differentiation of clinically similar benign and malignant tumors; 1. Diagnostic algorithm for keloids and hypertrophic scars, 2. Differential diagnosis of benign tumors that are similar in appearance to keloids and hypertrophic scars, 3. Differential diagnosis of malignant tumors that are similar in appearance to keloids and hypertrophic scars, 4. Clinical diagnosis of keloids and hypertrophic scars, 5. Pathological diagnosis of keloids and hypertrophic scars, 6. Imaging diagnosis of keloids and hypertrophic scars, and JSS 2015.

The second part is II. Treatment algorithms for keloids and hypertrophic scars at different medical facilities; 1. Medical treatment at general medical facilities, and 2. Medical treatment at specialized medical facilities.

The third part is III. Rationale behind each treatment for keloids and hypertrophic scars; 1. Topical adrenocortical hormone agent (administered by tape/plaster), 2. Adrenocortical hormone agent (administered by injection), 3. Other topical agents (corticosteroid and non-steroidal anti-inflammatory drug [NSAID] preparations, heparinoid ointment, and silicone gels and creams), 4. Oral medicines (tranilast, Saireito), 5. Rest/fixation therapy (administered by applying fixation tape or gel sheets), 6. Compression therapy (administered by applying bandages, supporters, garments, etc.), 7. Surgical excision and closure with simple sutures, 8. Surgical excision using the core excision method or partial resection, 9. Surgical excision followed by z-plasty, 10. Surgical excision followed by reconstruction with skin grafts or flaps, 11. Postoperative radiotherapy, 12. Radiation monotherapy, 13. Laser therapy, 14. Make-up therapy, 15. Psychosocial care, and 16. Other treatments (cryotherapy, 5-Fluorouracil (5-FU) injection, Botulinum toxin injection, and autologous fat grafting therapy).

The final part is IV. Site-specific treatment protocols; 1. Cartilaginous part of the auricle, 2. Earlobe, 3. Lower jaw, 4. Anterior chest wall (the scars developed from a midline chest incision), 5. Anterior chest wall (the scars developed from non-midline incisions or acne/folliculitis), 6. Upper arm, 7. Scapula, 8. Joint area (hand, elbow, knee, and foot), 9. Abdomen (the scars developed from an abdominal midline incision), 10. Abdomen (the scars developed from non-midline incisions), 11. Suprapubic, and 12. Other body areas.

In addition, the Consensus Document reveals the areas that require further scientific evidence or exploration. As such, it allows clinicians and scientists to identify the key research targets that will most effectively promote the accurate diagnosis and successful treatment of keloids and hypertrophic scars in the future.

## Main context

### Diagnostic algorithm for pathological scars and differentiation of clinically similar benign and malignant tumors

**Fig. 1:**
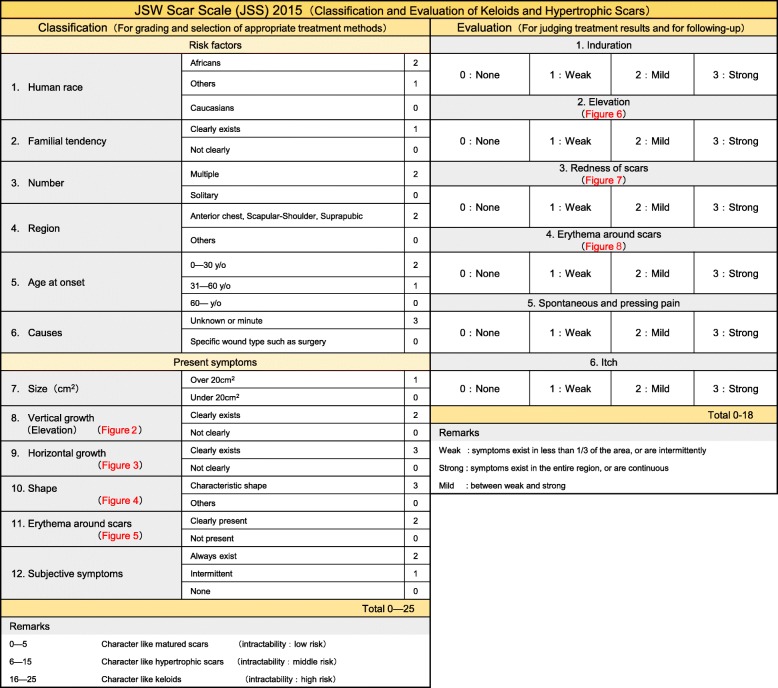
JSW Scar Scale (JSS)

**Fig. 2:**
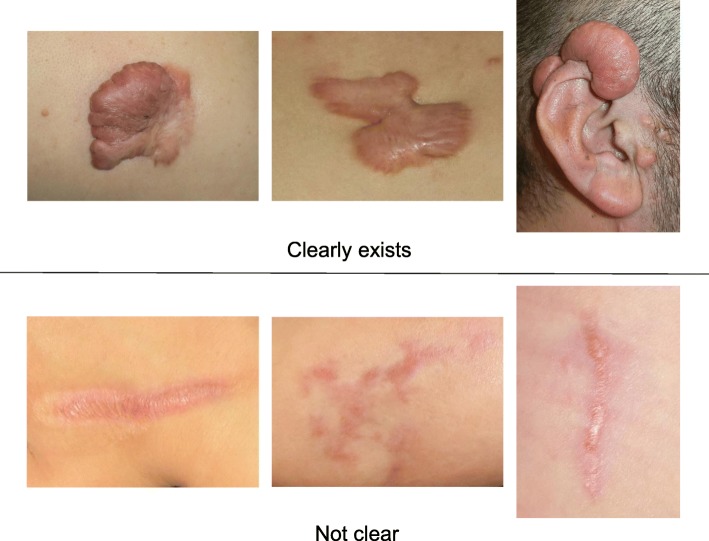
Vertical growth (elevation)

**Fig. 3:**
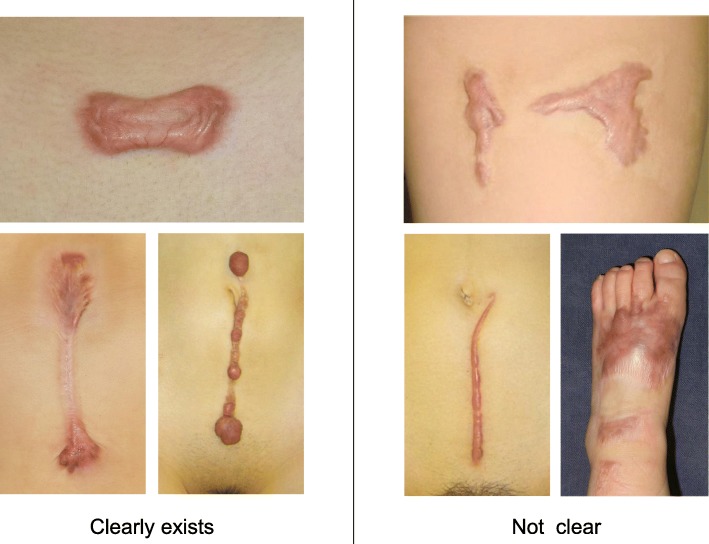
Horizontal growth

**Fig. 4:**
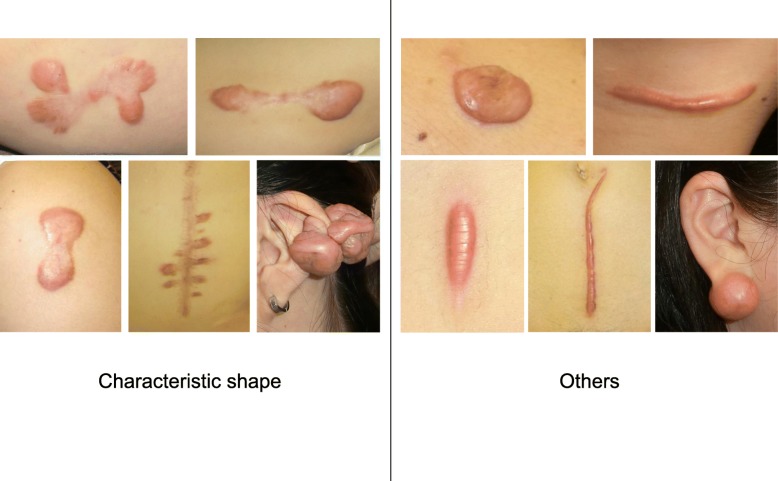
Shape

**Fig. 5:**
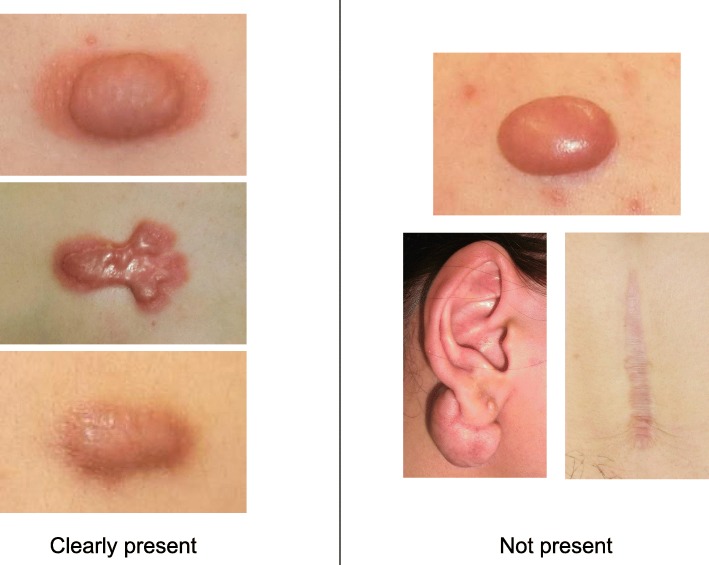
Erythema around scars

**Fig. 6:**
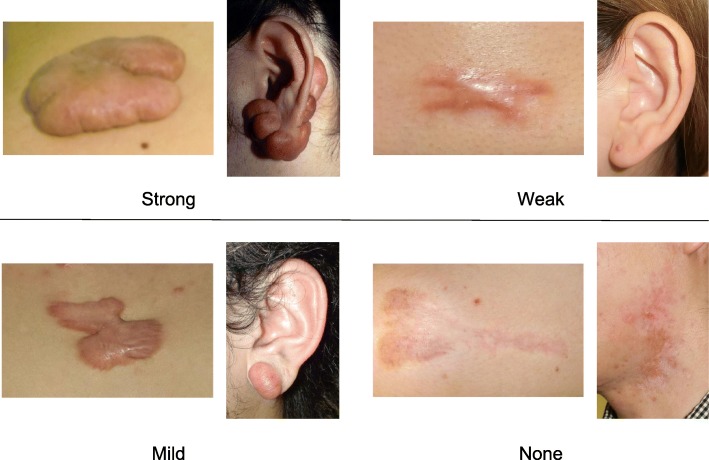
Elevation

**Fig. 7:**
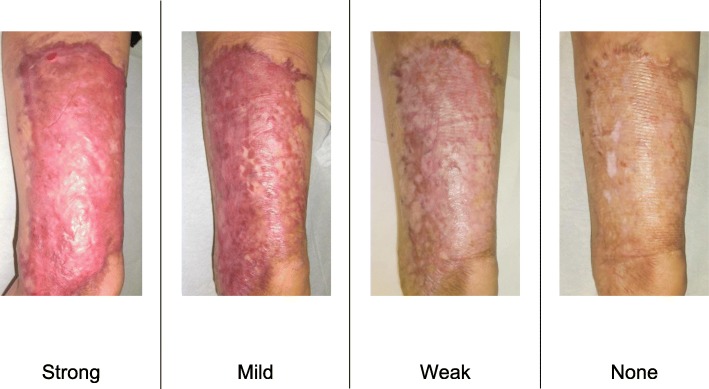
Redness of scars

**Fig. 8:**
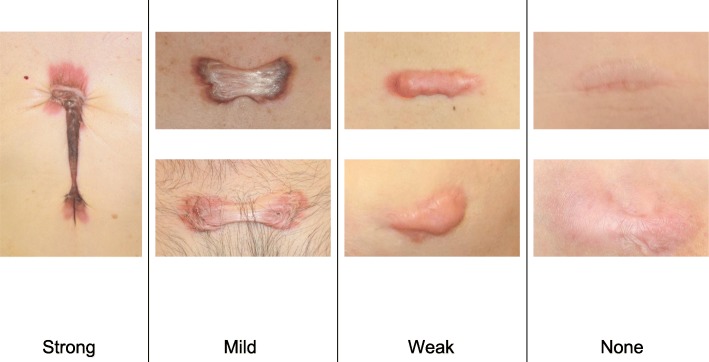
Erythema around scars

**Fig. 9:**
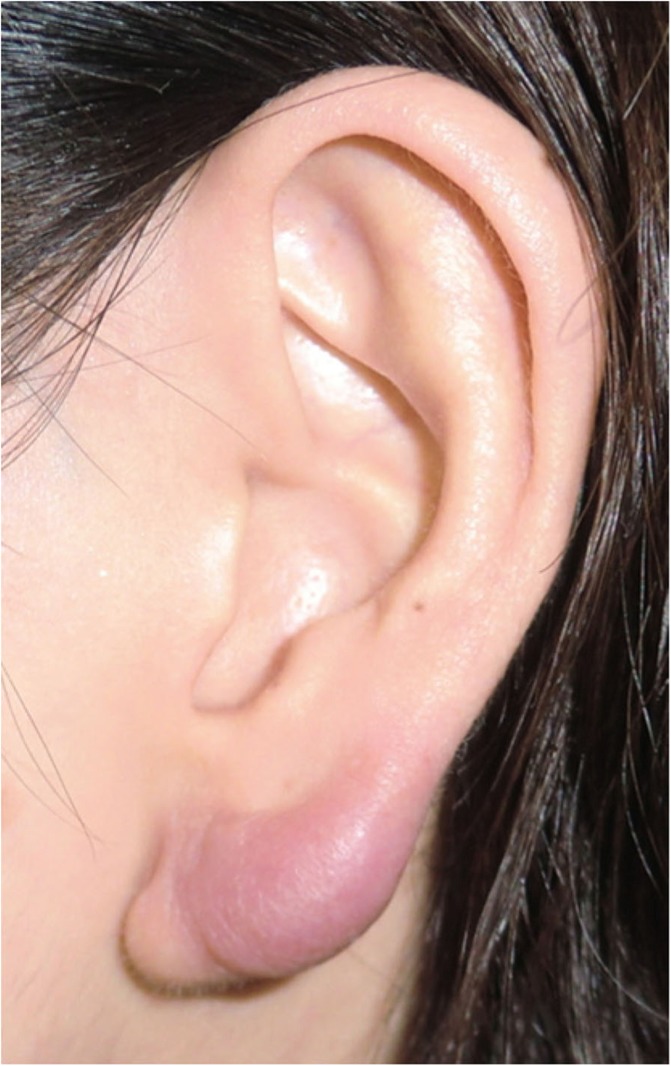
Pseudolymphoma

**Fig. 10:**
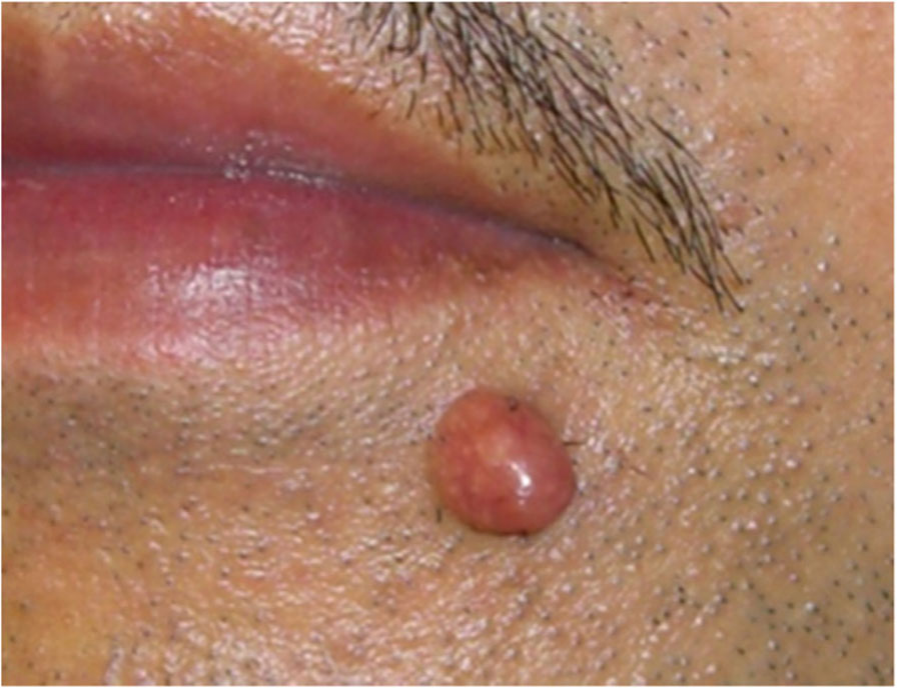
Mixed tumor of the skin

**Fig. 11:**
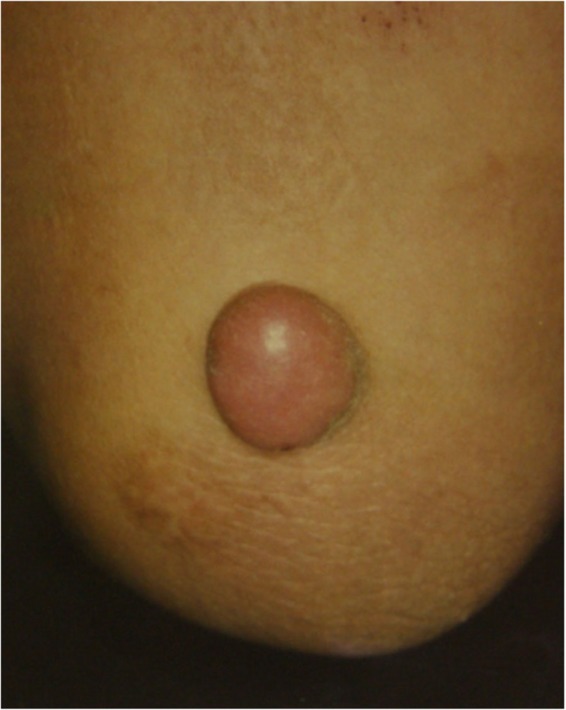
Xanthogranuloma

**Fig. 12:**
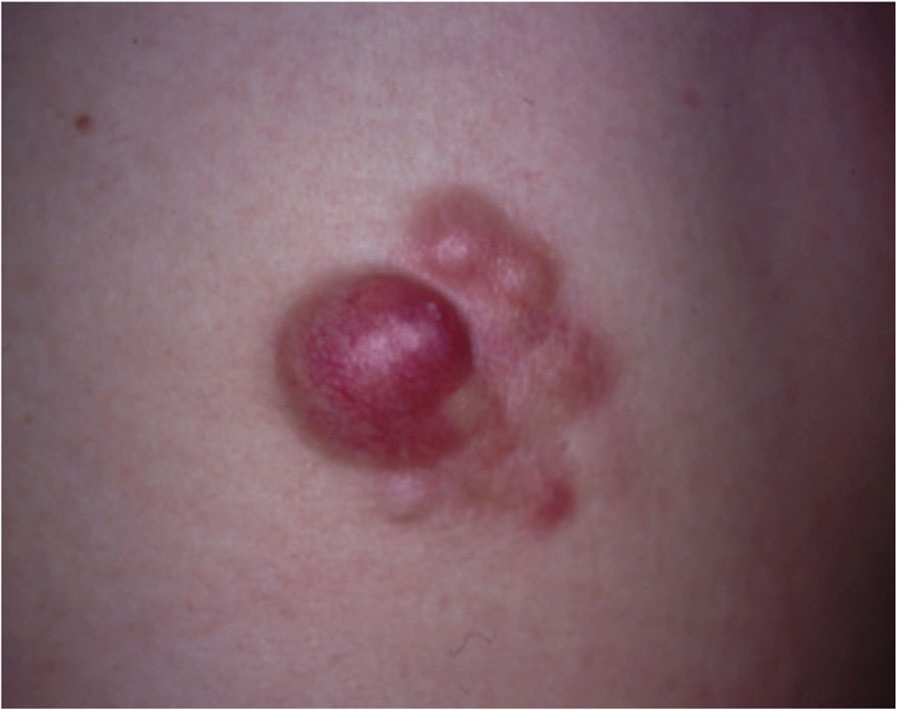
Dermatofibrosarcoma protuberans (DFSP)

**Fig. 13:**
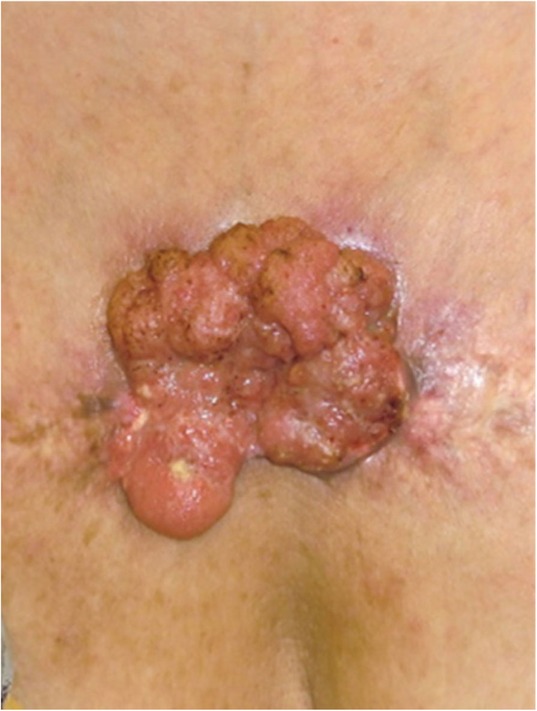
Squamous cell carcinoma (SCC)

**Fig. 14:**
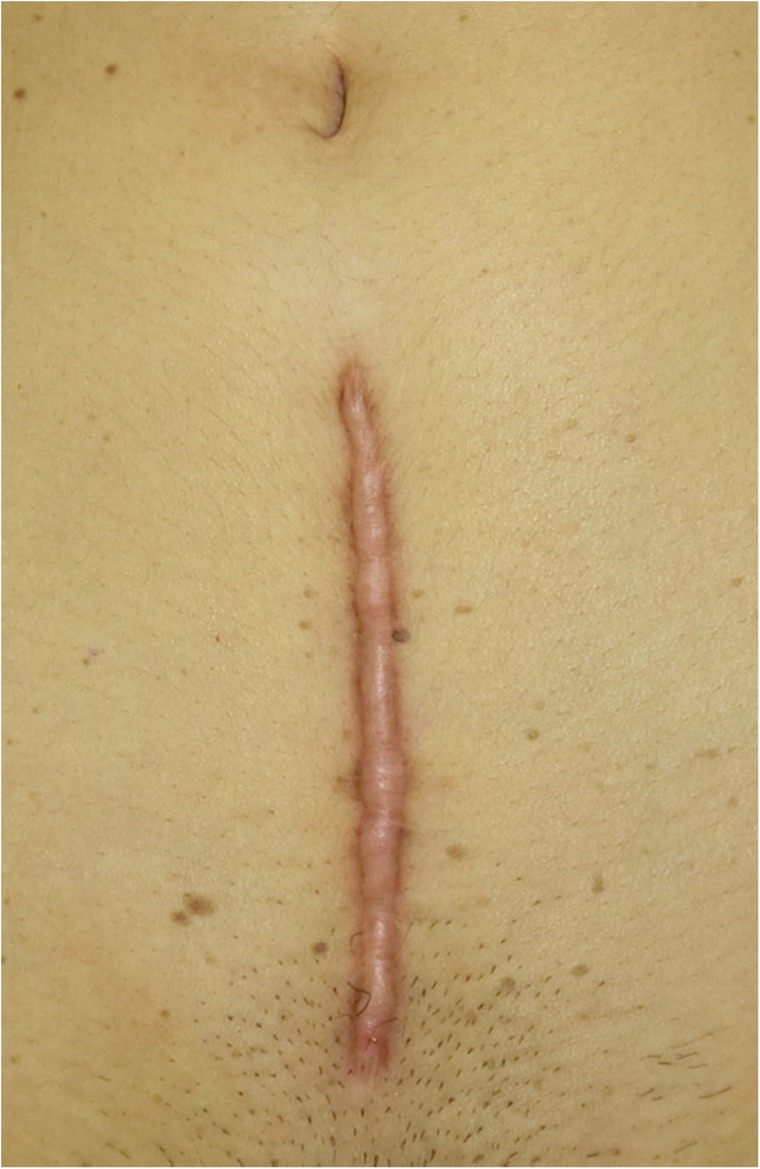
Typical hypertrophic scar

**Fig. 15:**
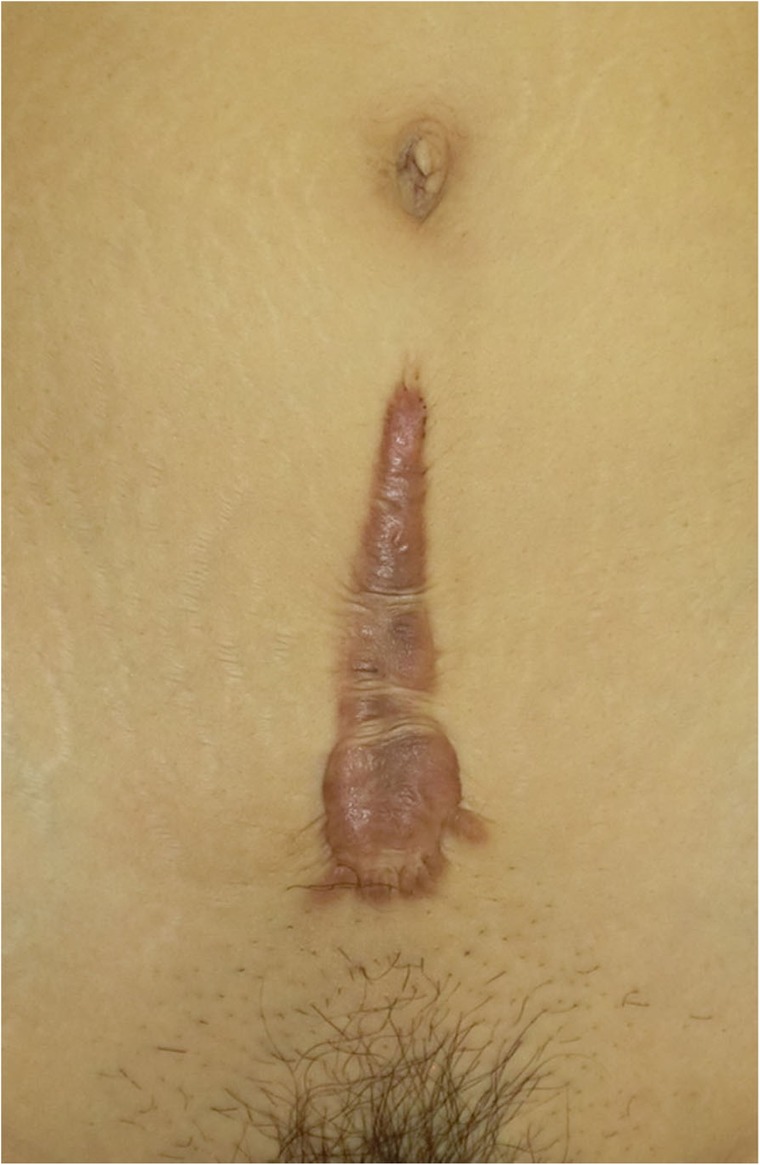
Intermediate lesion

**Fig. 16:**
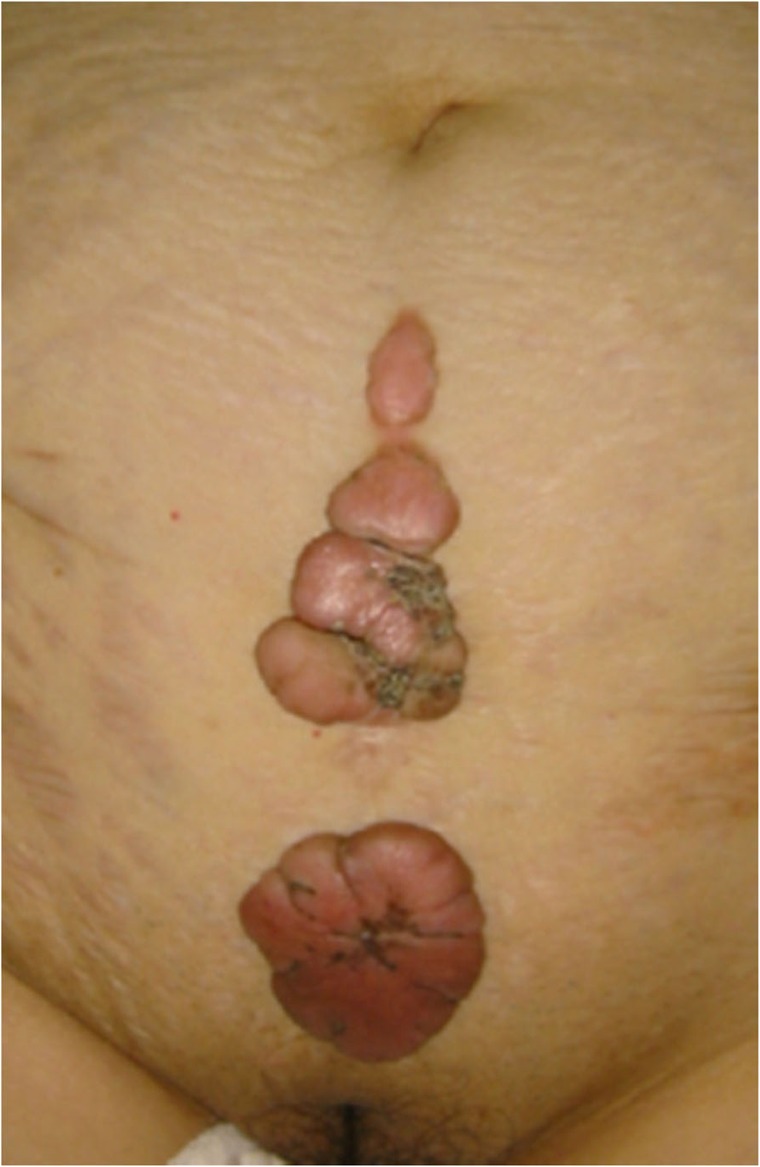
Typical keloid

**Fig. 17:**
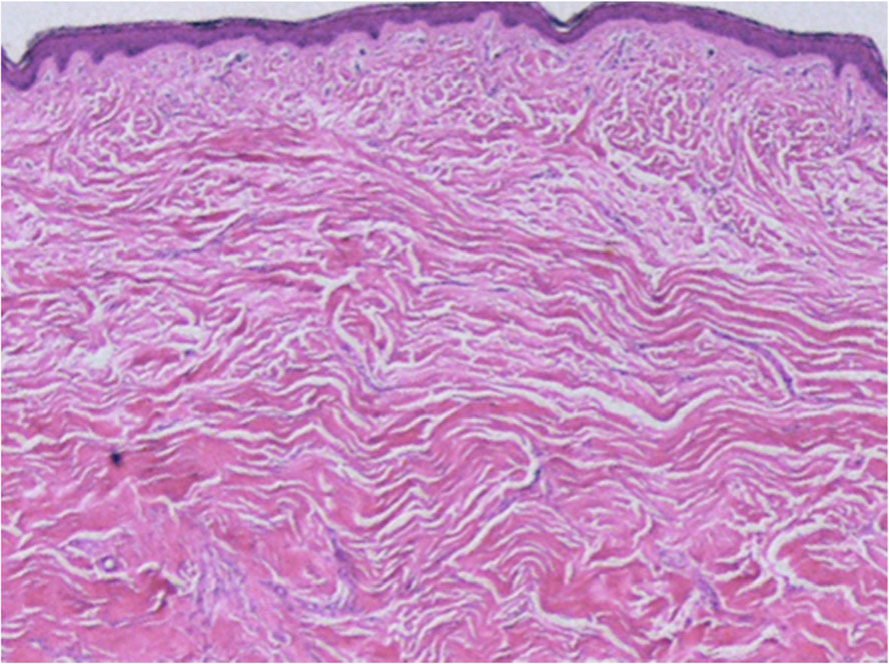
Typical hypertrophic scar (HE staining)

**Fig. 18:**
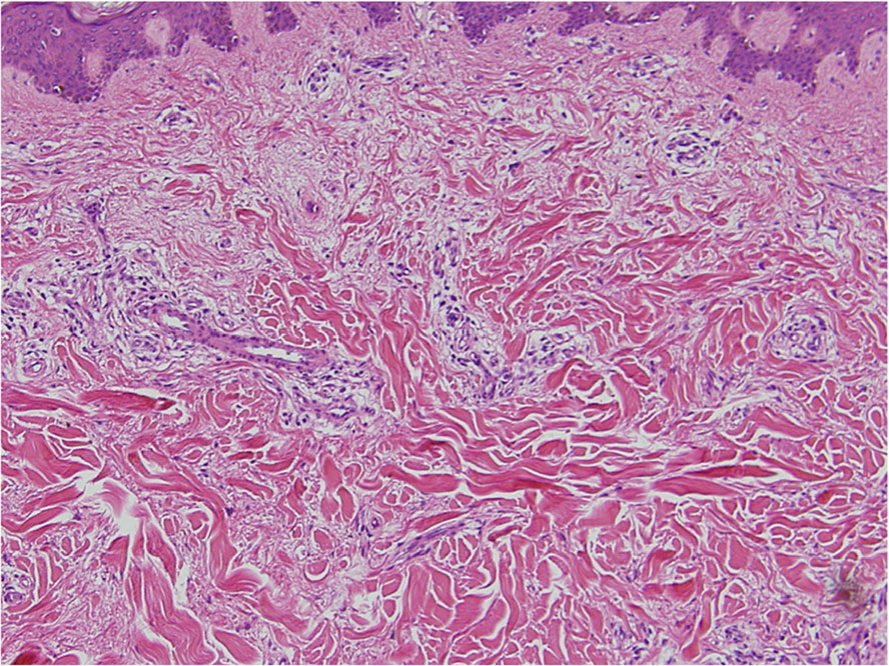
Typical keloid (HE staining)

1. Diagnostic algorithm for keloids, hypertrophic scars, and mature scars
  To determine whether the lesion is more likely to be a keloid [1], hypertrophic scar, or mature scar, score it according to the JSS 2015 classification (Figs. 1, 2, 3, 4, 5, 6, 7 and 8) [2].
  Mature scars have a JSS 2015 score of 5 or less [2].
  Lesions that are more likely to be hypertrophic scars have a JSS 2015 score of between 6 and 15 points [2]. These lesions can be treated in general medical facilities because there is a high possibility that they will respond to treatment.
  Lesions that are more likely to be keloids have a JSS 2015 score of 16 points or more [2]. It is advisable to treat these lesions in specialized medical facilities because there is a high possibility that they will be refractory to treatment (e.g., they may recur). Specialized medical facilities are facilities where patients with keloids and hypertrophic scars can be treated actively with multiple therapeutic measures.
2. Differential diagnosis of benign tumors that are similar in appearance to keloids and hypertrophic scars
  If the lesion is suspected to be a benign skin tumor rather than a keloid or a hypertrophic scar, a biopsy must be considered before treatment is implemented.
  The benign skin tumors that resemble keloids and hypertrophic scars are pseudolymphoma (Fig. 9), mixed tumor of the skin (Fig. 10), xanthogranuloma (Fig. 11), leiomyoma, and dermatofibroma.
3. Differential diagnosis of malignant tumors that are similar in appearance to keloids and hypertrophic scars
  Some malignant tumors are similar to keloids and hypertrophic scars in terms of their clinical features. If the lesion is suspected to be a malignant tumor (e.g., because its growth is rapid), it is essential to perform a biopsy.
  Dermatofibrosarcoma protuberans (DFSP) (Fig. 12), cutaneous squamous cell carcinoma (SCC) (Fig. 13), and amelanotic malignant melanoma sometimes present with clinical features that are similar to those of keloids and hypertrophic scars.
4. Clinical diagnosis of keloids and hypertrophic scars
  In general, hypertrophic scars do not grow outside the area of the original wound, whereas keloids grow laterally beyond the border of the wound [3]. Moreover, keloids and hypertrophic scars can differ in appearance (e.g., shape). This may reflect differences in the intensity and duration of the pathological inflammation that is suspected to drive the formation and progression of both lesion forms [4, 5]. However, in clinical practice, there are many lesions that exhibit intermediate growth and appearance characteristics that make it difficult to determine whether they are keloids or hypertrophic scars [4]. Examples of a classical hypertrophic scar, a classical keloid, and a difficult-to-diagnose intermediate lesion are shown in Figs. 14, 15, and 16.
  The JSS 2015 classifies lesions according to the clinical features discussed above along with the presence of risk factors such as early age of onset [2]. Lesions with a JSS 2015 score of 6 to 15 points have strong hypertrophic scar properties and respond well to treatment. By contrast, lesions with JSS 2015 scores of 16 points or more have strong keloid properties and tend to resist treatment. Thus, the JSS 2015 classification appears to reflect clinical reality.
  Biomarkers that can clearly distinguish keloids from hypertrophic scars have not yet been found, despite the many studies that have searched for them [6].
  Systemic factors can influence keloid and hypertrophic scar progression: both lesion forms are known to worsen in pregnant women [7, 8] and in patients with hypertension [9]. The lesions are also exacerbated by conditions that increase the levels of inflammatory cytokines, including IL-6 in the blood [10]. Conversely, empirical observations have shown that keloids and hypertrophic scars improve when pseudomenopausal therapy for such as uterine fibrosis and endometoriosis is instituted.
  Local stretching that places the skin under tension exacerbates keloids and hypertrophic scars [11]. This is reflected by the fact that both lesion forms tend to grow in the predominant direction of skin tension. Moreover, the pathological lesions of manual workers and athletes who repetitively perform a specific movement tend to be highly refractory and therefore require an extended treatment period.
5. Pathological diagnosis of keloids and hypertrophic scars
  In both keloids and hypertrophic scars, the epidermis and the papillary dermis have an almost normal structure (Figs. 17 and 18).
  Hypertrophic scars are characterized by dermal nodules that are composed of increased numbers of collagen bundles that run in different directions (Fig. 17). By contrast, keloids contain thick and uniformly stained collagen fibers that are called keloidal or hyalinized collagen. This keloidal collagen is mixed with dermal nodules that resemble those seen in hypertrophic scars [12, 13] (Fig. 18).
  At the histopathological level, keloids and hypertrophic scars can be distinguished on the basis of the degree of keloidal collagen. However, the absolute keloidal collagen threshold that accurately demarcates between the two lesion forms is not known. Therefore, it is difficult to draw a clear line between the two lesion forms. Consequently, the clinical diagnosis may not agree with the pathological diagnosis [5].
  Another notable histopathological finding of typical growing keloids is that they exhibit strong inflammation in the dermis at the leading edge of the keloid, namely, at the junction where the lesion meets the healthy skin.
  Strongly drying and scratched and/or ruptured keloid and hypertrophic scar tissue may be accompanied by hypertrophy of the horny layer of the epidermis and inflammation of the superficial dermis.
6. Imaging diagnosis of keloids and hypertrophic scars
  Keloids and hypertrophic scars can often be diagnosed by visual inspection and/or palpation. However, if a benign or malignant tumor is suspected, it is recommended to perform imaging diagnosis with ultrasound, CT, or MRI. These images can serve as a reference for the subsequent excisional biopsy, whose pathological analysis will lead to a definite diagnosis [1].
  However, if benign or malignant tumor is not suspected, it is better to subject the keloid/hypertrophic scar to ultrasonic elastography or ultrasound imaging because these imaging modalities indicate the hardness and other physical properties of the lesion in a noninvasive manner [14–16].
  When ultrasonic elastography is used for diagnosis, keloids and hypertrophic scars are depicted as harder areas than the surrounding tissues [14–16].
  Ultrasound imaging is also suitable for evaluating the effect of treatment on the keloid/hypertrophic scar over time [14–16]. This imaging modality depicts keloids and hypertrophic scars as low echo areas compared to the surrounding dermis. The inside of the lesion is often heterogeneous.
  At this stage, the existing diagnostic imaging modalities cannot readily distinguish keloids and hypertrophic scars from other similar benign tumors. Imaging modalities also do not accurately distinguish between keloids and hypertrophic scars.

### Treatment algorithms for keloids and hypertrophic scars at different medical facilities

**Fig. 19:**
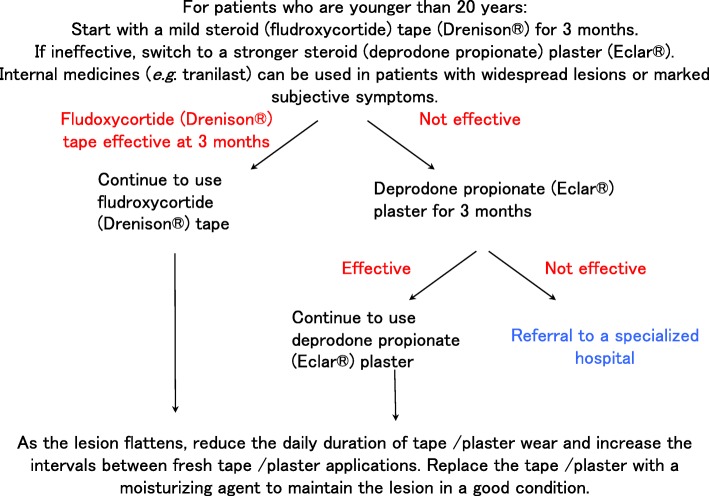
Keloid and hypertrophic scar treatment algorithm for pediatric patients

**Fig. 20:**
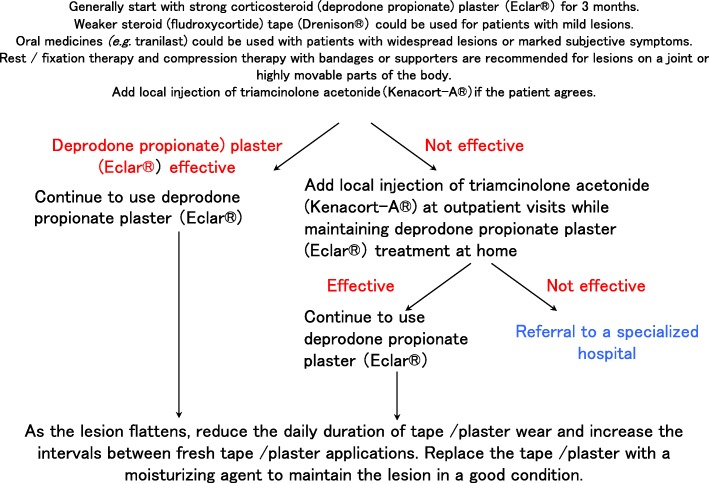
Keloid and hypertrophic scar treatment algorithm for adult patients

1. Medical treatment at general medical facilities (Table 1)
  After definitively diagnosing the lesion as a keloid or hypertrophic scar, it is recommended that pediatric patients undergo continuous treatment with corticosteroid tape/plaster, as detailed in Fig. 19. A weak steroid tape should be tried first for 3 months. If it is not effective, a stronger steroid plaster should be tried for another 3 months. Oral medicines such as tranilast can be given for severe cases. If the steroid tape/plaster treatment is not effective, the patient should be referred to a specialized medical facility.
  In terms of adult patients, it is recommended that they start immediately with strong steroid plasters for 3 months (although a weak steroid tape can use used in mild cases). If the plasters are not effective, triamcinolone acetonide injections can be added. Oral medicines such as tranilast can be given for severe cases. Rest/fixation and compression therapies should be provided if the lesion is on a joint or highly movable body site. If the steroid plaster and injections are not effective, the patient should be referred to a specialized medical facility (Fig. 20).
  Patients with keloids or hypertrophic scars should be encouraged to improve lifestyle habits that may contribute to scar exacerbation. These lifestyle habits include physical labor or excessive exercise that involves repeating a motion that places tension on the scar.
  Other more general points to consider are measures that prevent keloids and hypertrophic scars from arising in the first place. First, when patients in general present with a wound, it is recommended to carefully clean and disinfect the wound, apply topical antibiotics as needed, and strap the wound with fixation material that protects it from local stretching forces. This approach should be taken even if the wound is small and mild because hypertrophic scars and especially keloids can develop from apparently inconsequential wounds.
  Second, with all patients, the treatment for their particular problem should start with the least invasive option.
2. Medical treatment at specialized facilities (Table 2)
  Make a definitive diagnosis of keloid or hypertrophic scar, evaluate the subjective symptoms, and note the location of the lesions.
  Select the most appropriate treatment strategy on the basis of the location of the lesions. The site-specific treatment regimens are presented in “Site-specific treatment protocols” section. These regimens involve multiple therapies, including external and oral medicines, rest/fixation and compression therapies, surgery, radiotherapy, laser therapy, make-up therapy, and psychosocial health care. The rationale behind each therapy is presented in “Rationale behind each treatment for keloids and hypertrophic scars” section.
  Determine whether the patient has lifestyle habits that can exacerbate scar growth. These lifestyle habits include physical labor and excessive exercise that involves repeating a motion that places tension on the scar. If the patient has such lifestyle habits, he/she should be encouraged to improve them.

**Table 1:** Keloid and hypertrophic scar treatment algorithm for general medical facilities

Corticosteroid tape/plaster, ointment Various external medicines Oral medicines; e.g., tranilast, Saireito extract Rest / fixation therapy; e.g., taping, silicone gel sheeting Compression therapy; e.g., bandages, supporters, garments

**Table 2:** Keloid and hypertrophic scar treatment algorithm for specialized medical facilities

Corticosteroid tape/plaster, ointment, injections Various external medicines Oral medicines; e.g., tranilast, Saireito extract Rest / fixation therapy; e.g., taping, silicone gel sheeting Compression therapy; e.g., bandages, supporters, garments Surgery Radiotherapy Laser therapy Make-up therapy Psychosocial health care Others	

### Rationale behind each treatment for keloids and hypertrophic scars

**Fig. 21:**
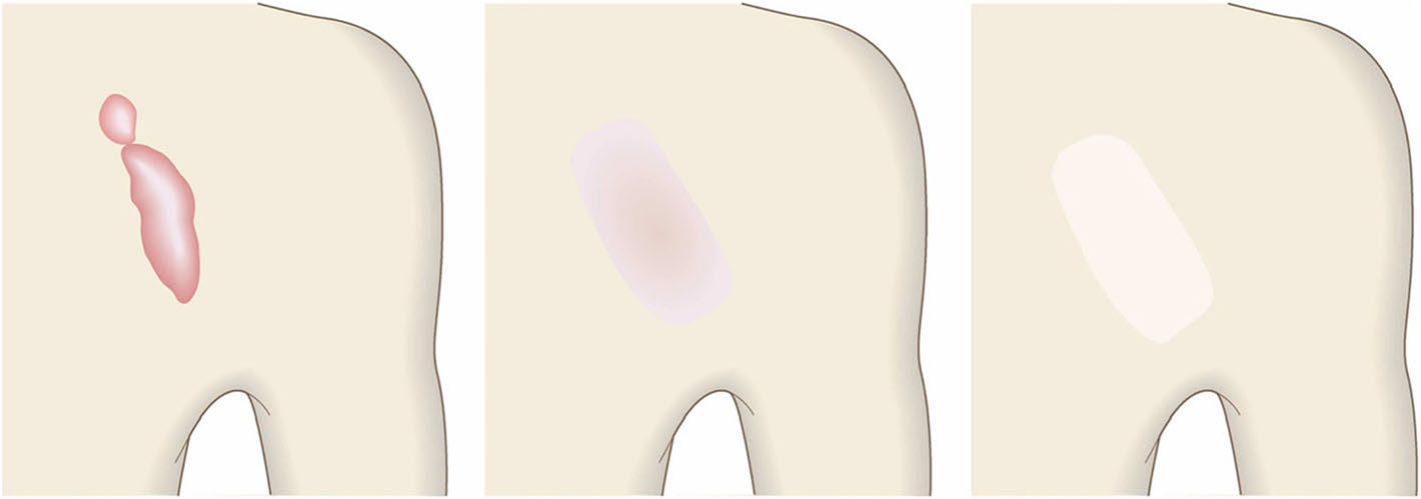
Topical adrenocortical hormone agent (administered via tape/plaster). When the corticosteroid tape/plaster therapy starts to improve the height and stiffness of the keloid/hypertrophic scar, the tape/plaster area being used, the affixation duration, and the intervals between fresh applications should be reduced gradually

**Fig. 22:**
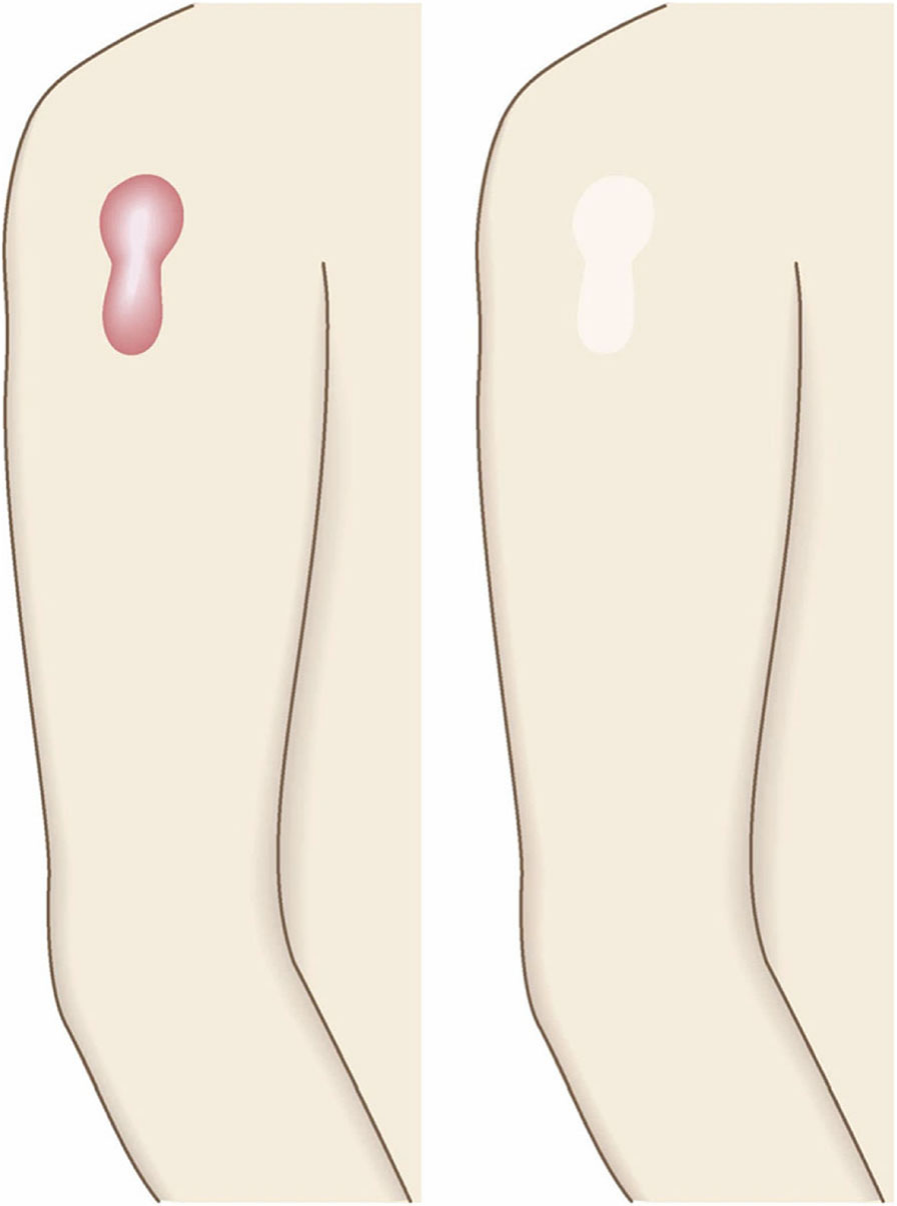
Local adrenocortical hormone agent (administered by injection). Corticosteroid injections rapidly improve the symptoms of keloids and hypertrophic scars but their drawback is injection-induced pain. Means to prevent this pain should be implemented

**Fig. 23:**
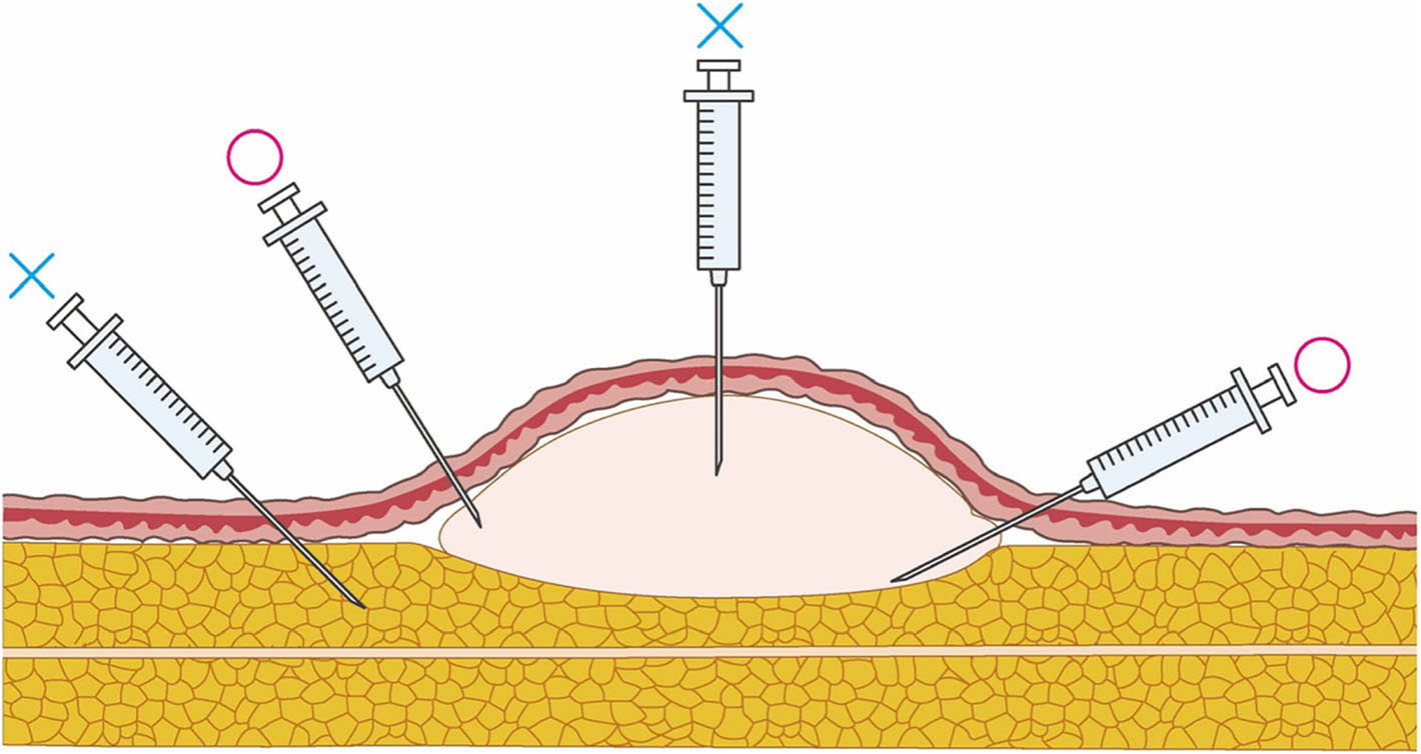
The target of the injection. When injecting pathological scars with corticosteroid, do not inject the solid central fibrotic mass of the lesion because the drug will not infiltrate the tissue adequately. Moreover, the rising pressure induced by the injection may cause pain. Instead, penetrate the scar from its border with the normal skin. The target is the deepest part of the scar and/or the periphery of the scar, where the inflammation is particularly pronounced

**Fig. 24:**
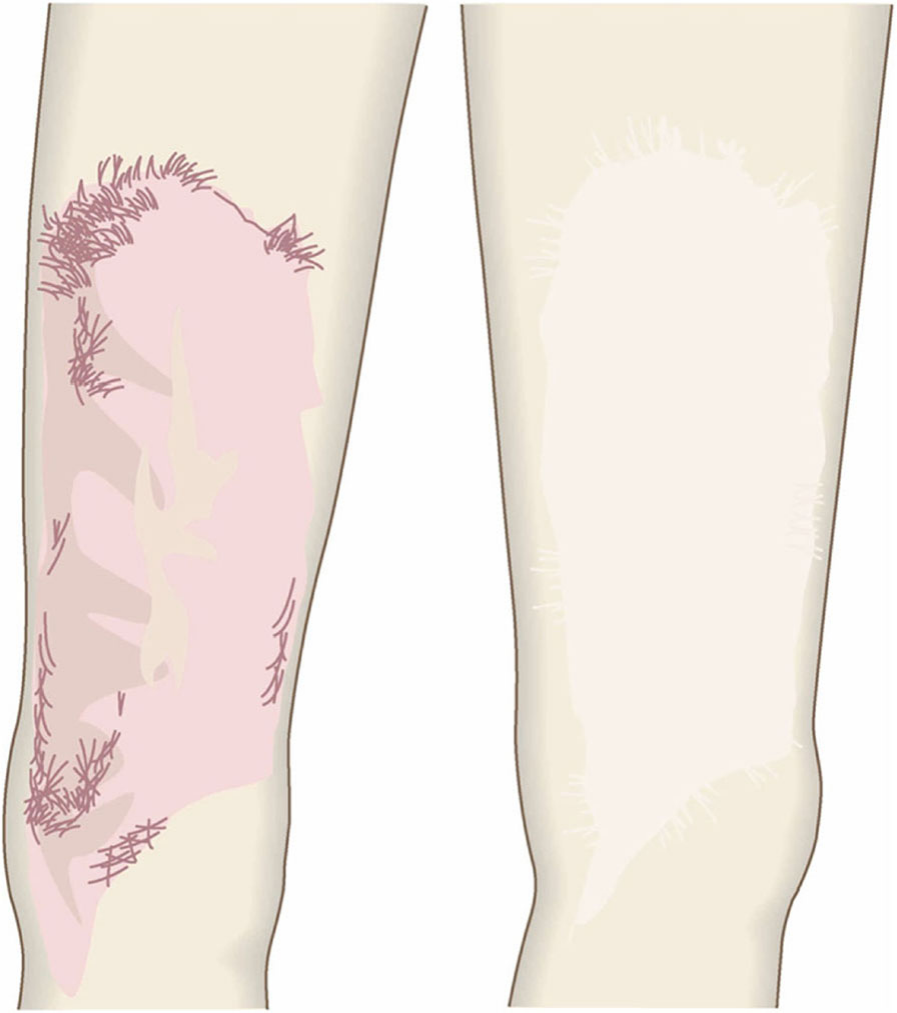
Topical agents (corticosteroid and non-steroidal antiinflammatory drug [NSAID] preparations, heparinoid ointment, and silicone gels and creams). Treatment with topical preparations such as corticosteroid, NSAID, heparinoid, and silicone ointments, gels, and creams reduce inflammation. The goal is to induce scar maturation. However, the shape of the scar will remain after maturation

**Fig. 25:**
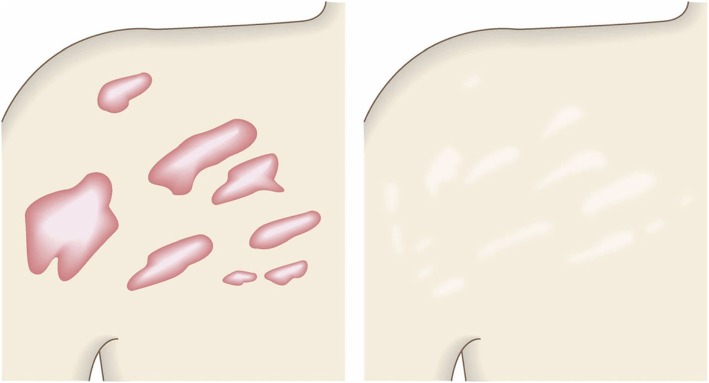
Oral medicines (tranilast, Saireito). It is recommended to use oral medicines when the patient has huge and/or multiple keloids or hypertrophic scars, since these conditions suggest the presence of a systemic risk factor

**Fig. 26:**
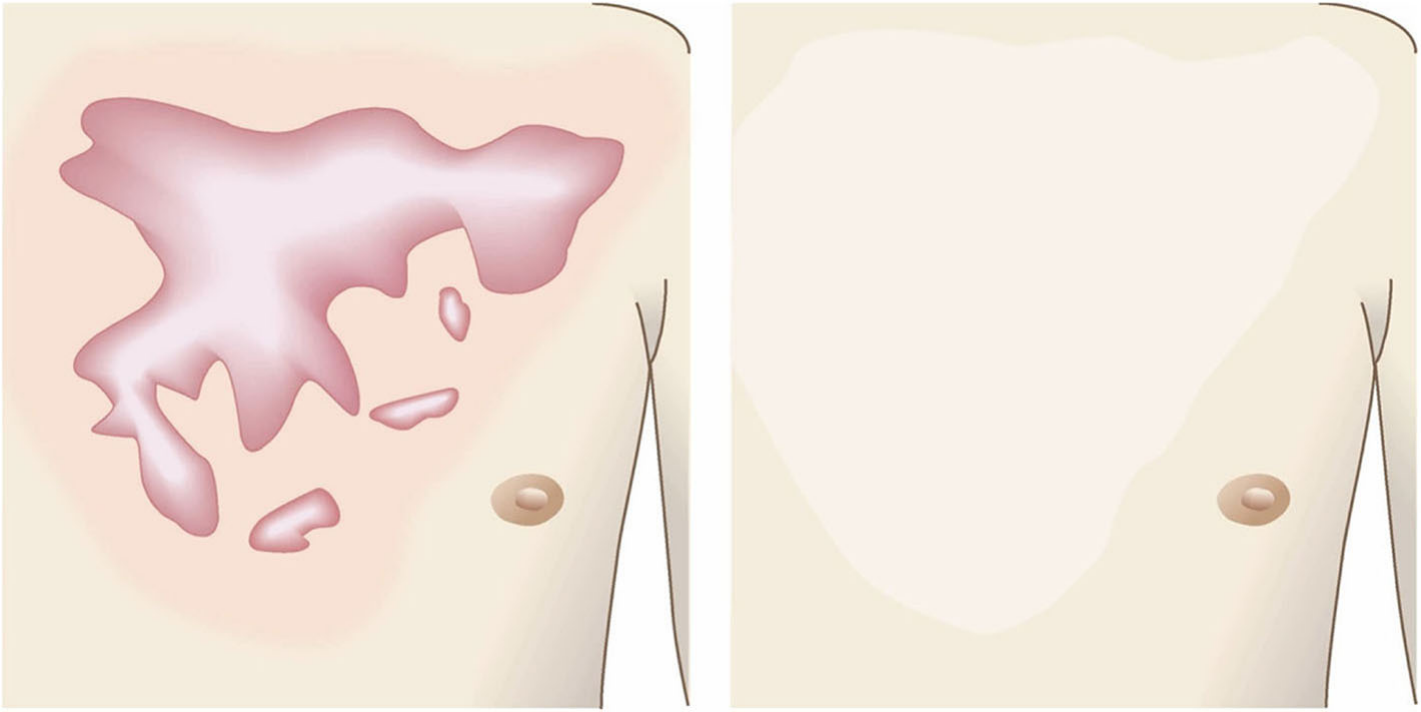
Rest/fixation therapy (administered by applying fixation tape or gel sheets). Fixation tape and gel sheets can reduce the tension on the pathological scar, thereby promoting scar maturation

**Fig. 27:**
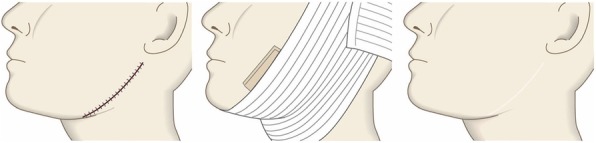
Compression therapy (administered by applying bandages, supporters, garments, etc.). Compression therapy acts by placing pressure on the blood vessels around and in the keloid/hypertrophic scar. This reduces the blood flow in the lesion, which in turn suppresses scar inflammation and promotes scar maturation

**Fig. 28:**
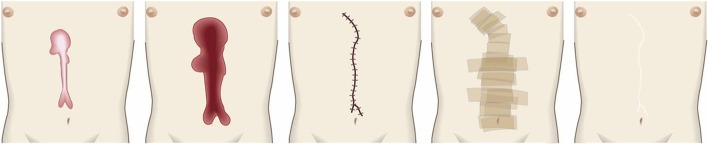
Surgical excision and closure with simple sutures. When excising keloids or hypertrophic scars from body sites that have strong skin tension (e.g., the trunk), the fatty tissues should be removed along with the scar. The fasciae should then be undermined. Thereafter, the fasciae should be sutured so that the upper layers of the skin approximate each other closely. This makes it easy to place dermal sutures with minimal tension

**Fig. 29:**
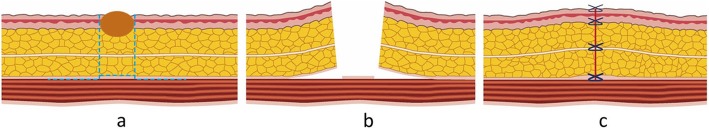
The ideal suture method. **a** The scar should be removed along with the fatty tissue under the scar. **b** Undermine below the deep fascia of the muscle and then suture first the deep fasciae and then the superficial fasciae. **c** This suturing strategy causes the upper skin layers to attach to each other naturally. Dermal sutures can then be started

**Fig. 30:**
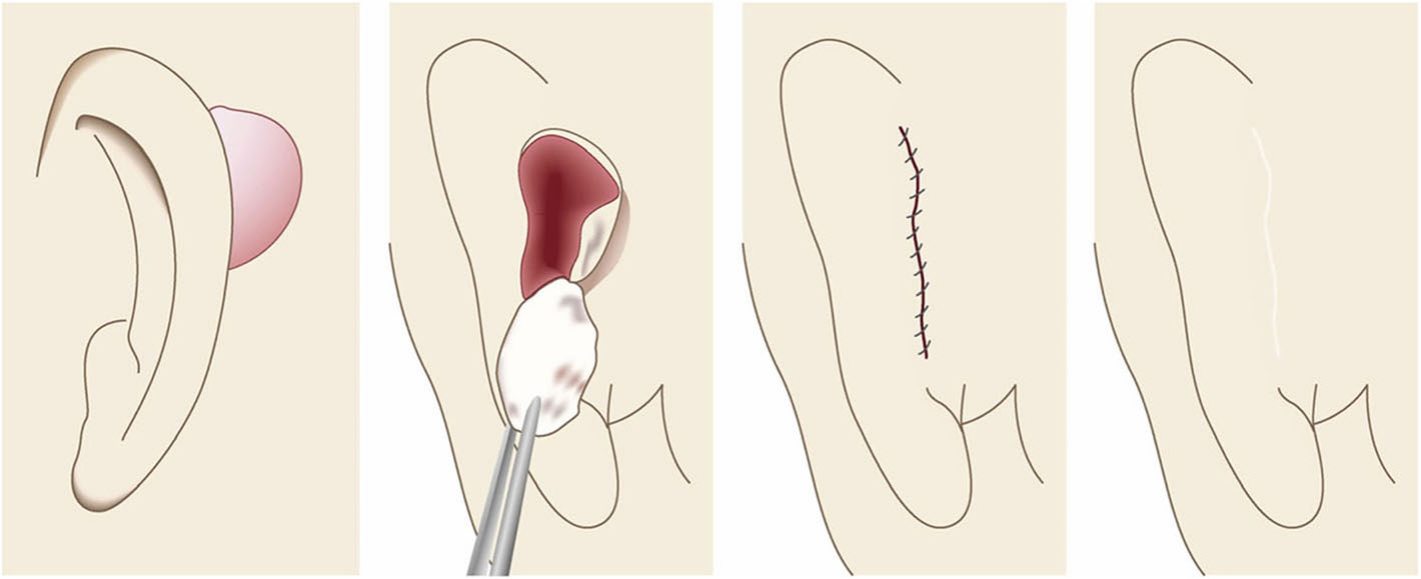
Surgical excision using the core excision method or partial resection. If the lesion is large or if total removal might result in significant deformity, it is recommended to remove only the fibrous core of the keloid/hypertrophic scar

**Fig. 31:**
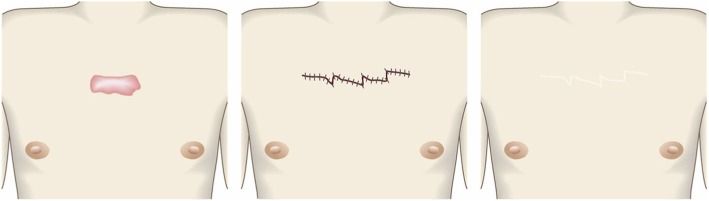
Surgical excision followed by z-plasty. If the incision line used to excise a keloid/hypertrophic scar follows the predominant direction of skin tension, z-plasty should be applied. This will disperse the tension on the wound/scar

**Fig. 32:**
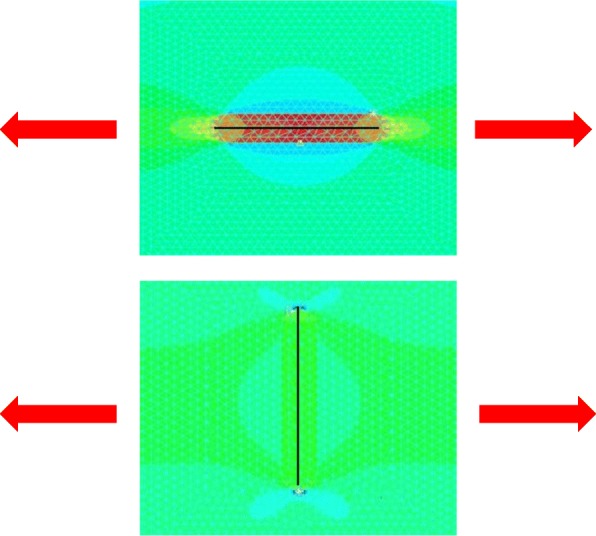
Computer simulation of the effect on wound tension. Computer simulation of the effect on wound tension when the directions of the incision and the predominant skin tension do and do not coincide. **a** When the incision line follows the direction of skin tension, the tension on the entire length of the wound will be high during the wound healing process (red color in the upper panel). **b** If the incision lies perpendicular to the direction of skin tension, the force will be dispersed along the wound and less tension will be placed on the wound (green color in the lower panel)

**Fig. 33:**
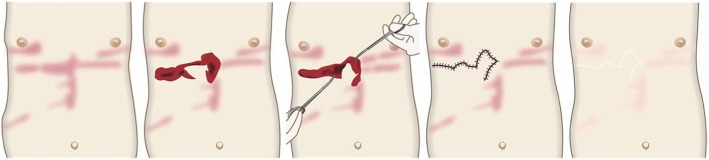
Surgical excision followed by reconstruction with skin grafts or flaps. If primary closure after keloid/hypertrophic scar excision cannot be performed with low tension, it is best to consider reconstruction with skin grafts or flaps. The procedure should be followed with postoperative adjuvant therapies such as radiotherapy

**Fig. 34:**
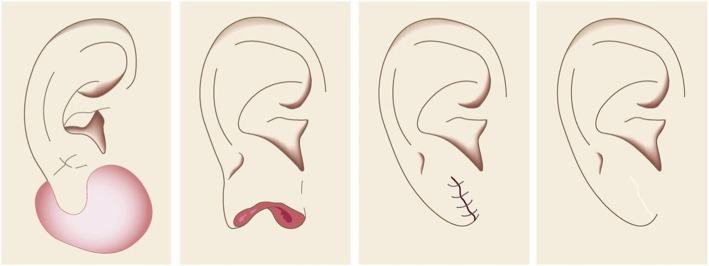
Postoperative radiotherapy. Body sites differ in terms of the postoperative radiotherapy protocol that is needed to prevent recurrence after keloid/hypertrophic scar excision. For example, earlobe keloid surgery should be followed with 10 Gy/2 fractions/2 days radiotherapy

**Fig. 35:**
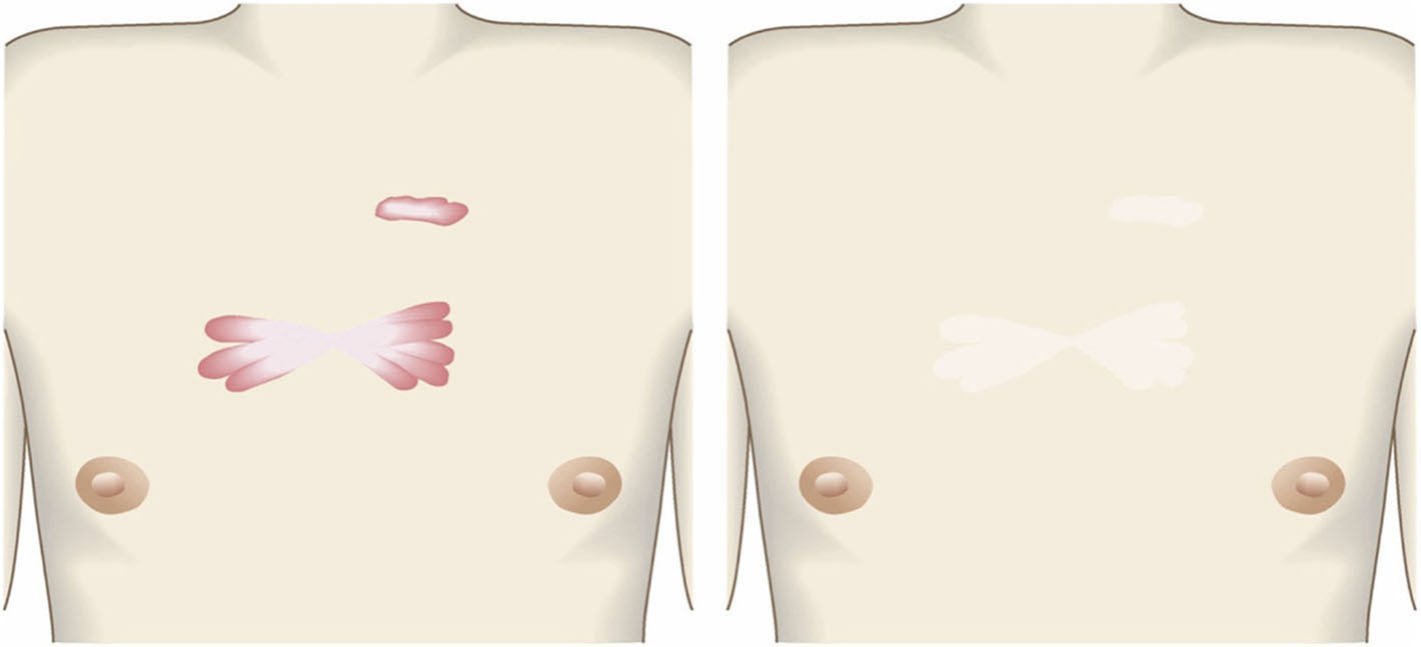
Radiation monotherapy. Radiation monotherapy may be suitable for the few cases in which surgery will be difficult to perform. The radiation monotherapy can improve the severe pain and itch of the keloid/hypertrophic scar

**Fig. 36:**
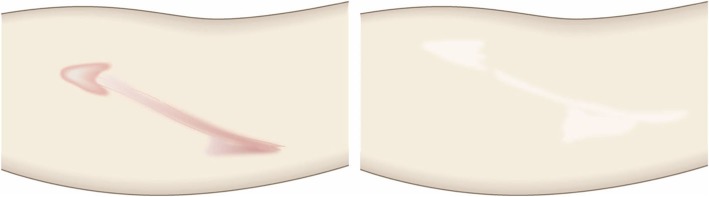
Laser therapy. Laser therapy can improve the color of keloids and hypertrophic scars. Flat scars are particularly indicated for laser therapy

**Fig. 37:**
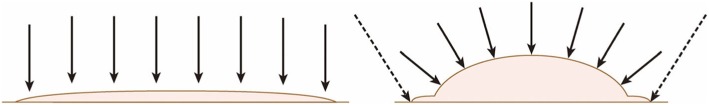
The ideal irradiation method of lasers. When performing laser therapy, the laser beam (arrows) should be held perpendicularly to the scar surface. This 90° orientation should be maintained as the beam is passed over the curvature of the scar. When irradiating the boundary of the scar (dashed arrows), it is permissible to irradiate some of the adjacent normal skin

**Fig. 38:**
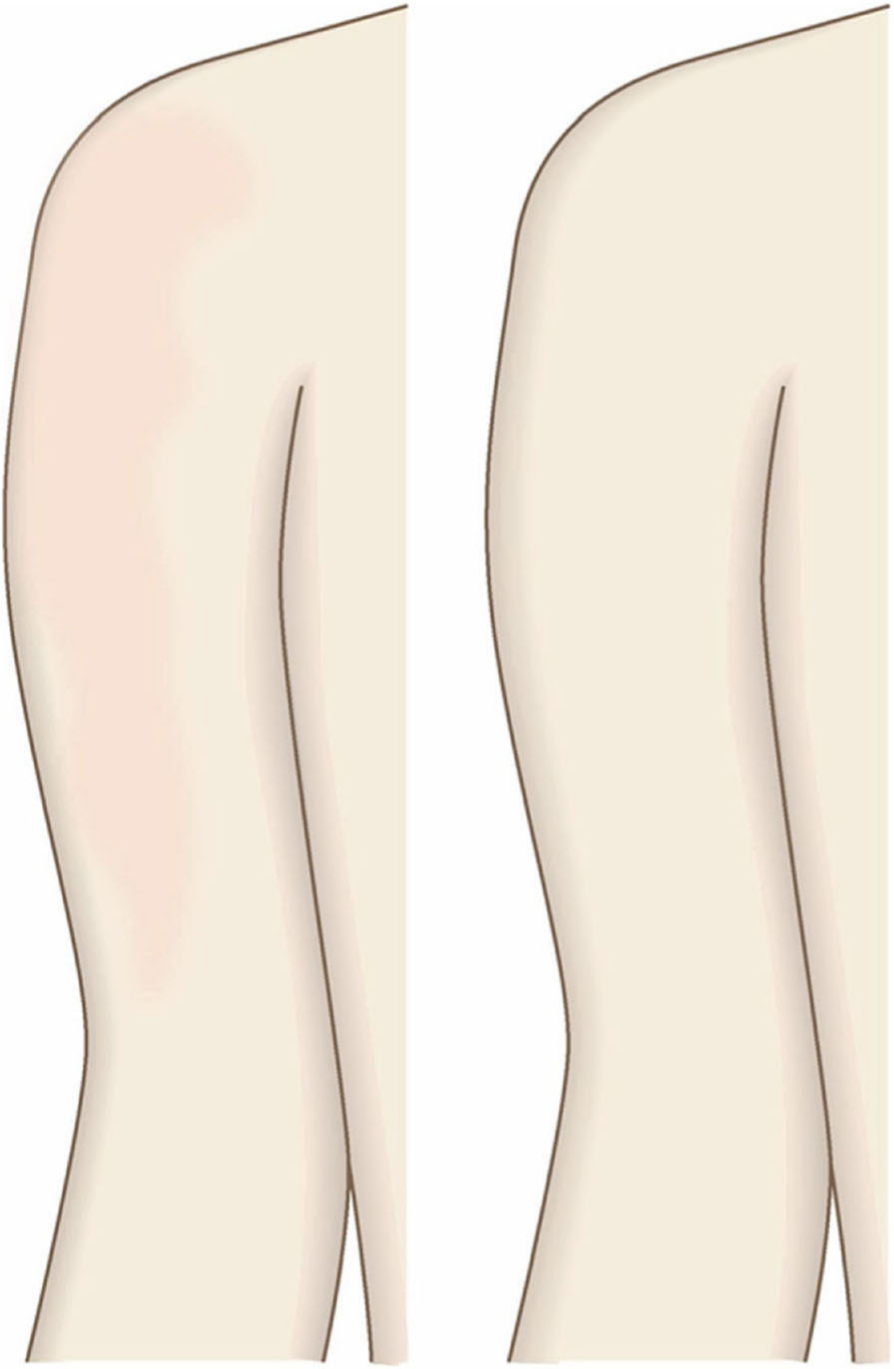
Make-up therapy. Medical make-up therapy can temporarily improve the appearance of keloids, hypertrophic scars, and mature scars. This can improve the mental health of the patient

1. Topical adrenocortical hormone agent (administered via tape/plaster) (Fig. 21)
  Concept
    There are two types of tape/plaster that deliver topical corticosteroid. They differ in terms of corticosteroid strength: the stronger one is the deprodone propionate (Eclar®) plaster while the weaker one is the fludroxycortide (Drenison®) tape [17–20].
    Topical corticosteroid preparations fall into one of five potency grades. The Eclar® pdeprodone propionate plaster belongs to the third grade (strong) while the Drenison® fludroxycortide tape is considered to be a fourth grade preparation (medium). However, since these preparations are applied to keloids and hypertrophic scars with the occlusive dressing technique (ODT), the effect size of both is expected to be 1–2 grades stronger than usual.
    Since children have relatively thin skin, the first choice for pediatric patients should be the Drenison® tape. By contrast, the Eclar_®_ plaster is the first choice for adult patients. However, the tape/plaster choice also depends on lesion severity and side effects.
    The tape/plaster should be applied after cutting the adhesive material to match the shape of the scar: there should be little overlap onto the normal skin. The tape/plaster should initially be used continuously for 3 months. It should be changed every 24–48 h.
  Hints and tips
    After the steroid tape/plaster has caused the lesion to flatten and soften sufficiently, the hours affixed and the intervals between fresh tape/plaster applications should be gradually decreased. Eventually, the tape/plaster treatment should be replaced with steroids in external use preparations (e.g., ointments, gels, and creams) or ointments and creams that contain non-steroidal anti-inflammatory drugs (NSAIDs). Eventually, these preparations can be replaced by external medications that moisturize the skin (e.g., heparinoid ointment). All of these preparations will help to prevent inflammation from arising again.
    The patient should be instructed to start using the tape/plaster again if the lesion relapses or a new keloid or hypertrophic scar arises.
    If the patient is a child, the parents should be instructed to apply a fresh tape/plaster every day after the child takes a bath.
    While the tape/plaster should generally be cut according to the shape of the scar to avoid contact with normal skin, this may not be possible if there are multiple interconnecting lesions that make cutting the tape/plaster sheet cumbersome and time-consuming. In this case, single sheets that cover the scarred area should be applied.
  Attention
    Steroid tape/plaster can cause irritant contact dermatitis and allergic contact dermatitis [17, 19]. Irritant contact dermatitis can improve if the overall affixation duration is reduced. By contrast, if allergic contact dermatitis occurs, the tape/plaster can no longer used. In this case, other external medications such as NSAID creams should be considered. Steroid creams and ointments can also be used if the allergic contact dermatitis of the patient is due to the tape/plaster material or the adhesive rather than to the steroid.
    To prevent adverse effect on glaucoma or cataracts, tape/plaster affixation around the eyes should be avoided.
    If steroid acne appears, the acne should be treated simultaneously or the treatment should be halted temporarily.
    If the skin of the lesion appears to be getting thinner and there are indications of telangiectasia, the tape/plaster should be replaced with external moisturizing preparations such as heparinoid ointment. The tape/plaster treatment should also be halted if the scar is still “red” but it has flattened completely and has softened to the point that palpation can no longer determine its presence. This is because the redness could be due to capillary dilation rather than scar inflammation.
    It should be remembered that long-term use of large quantities of steroid in children can cause developmental impairment due to the effect of steroid on DNA synthesis and cell proliferation [21].
    Long-term steroid use over large areas should be avoided in pregnant women because animal experiments have shown that steroids can be teratogenic [22].
  Goal
    The goal of this treatment is to induce the scar to flatten and soften. The redness of the lesion can remain: it will generally improve after the tape/plaster applications are stopped.
2. Local adrenocortical hormone agent (administered by injection) (Fig. 22)
  Concept
    The corticosteroid in this case is triamcinolone acetonide (Kenacort-A®) [23–26]. Injections with this agent can be used to either ameliorate existing keloids and hypertrophic scars [23, 24] or to prevent relapse after excisional surgery [25, 26].
  Hints and tips
    It is recommended that each injection should consist of 2–5 ml of a triamcinolone acetonide preparation that is generated by diluting 5–10 mg with a local anesthetic such as xylocaine 1% with epinephrine. Women may experience fewer menstrual irregularities if the triamcinolone acetonide dose does not exceed 5 mg.
    Several reports have described measures that can prevent the pain caused by triamcinolone acetonide injection [27, 28]. They recommend to apply anesthetic tapes and creams. They also suggest injecting local anesthetic before proceeding with the triamcinolone acetonide injection [28]. However, they caution against injecting hard areas with large quantities of triamcinolone acetonide, even if the injection does not hurt at the time. This is because when the anesthesia wears off, the patient may experience strong pain.
    Thin needles such as 30G and 27G should be used along with syringes with locks.
    Initially, the target of the injection should not be the center of the hard mass of the keloid or hypertrophic scar. This is because the injection fluid will not infiltrate the tissue sufficiently. The rising pressure caused by injecting the hard mass may also cause pain. Instead, the needle should enter the scar from its border with the normal skin and target either the deepest part of the scar (because the deepest part of the scar is softer than its central core) or the most heavily inflamed part of the scar at the junction between the normal skin and the scar (Fig. 23).
    After the scar has softened, the needle can be injected straight into the core.
    Repeated injections should be spaced out by 2-week intervals.
    Smaller keloids and hypertrophic scars can improve markedly after just one or two injections. In this case, further injections are not needed if the improvement can be maintained by applying corticosteroid tape/plaster.
    The main advantage of the corticosteroid injection is the quickness of its effect on subjective symptom. However, precautions that prevent pain from the injection should be implemented.
  Attention
    Injecting fatty tissues with triamcinolone acetonide should be done with caution because it can cause the fatty tissue to atrophy.
    Pregnant women should not undergo triamcinolone acetonide injections. In addition, the injections should be avoided in patients with diabetes mellitus, glaucoma, or cataracts.
    The dose should be considered carefully because high doses can cause menstrual irregularities in women and lower bone density in elderly patients.
    In particular, lower doses should be used with children and elderly people because there are several reports of iatrogenic Cushing syndrome developing in both groups after triamcinolone acetonide injections [29–31].
    Injection around the face should be performed with caution because there was a case report of blindness caused by the triamcinolone acetonide embolus [32].
    If steroid acne is observed, the acne should be treated simultaneously or the treatment should be stopped temporarily.
  Goal
    The goal of this treatment is to induce the scar to flatten, soften, and mature.
3. Other topical agents (corticosteroid and non-steroidal anti-inflammatory drug [NSAID] preparations, heparinoid ointment, and silicone gels and creams) (Fig. 24)
  Concept
    All of these preparations help to suppress inflammation. Corticosteroids have the strongest effect, followed by NSAID [33]. Heparinoid ointment and silicone gels and creams also help to reduce inflammation and promote scar maturation by moisturizing the scar surface [33–35].
  Hints and tips
    Corticosteroid ointments and creams will not be as strong as 24-h corticosteroid tape/plaster application unless they are applied several times a day with the occlusive dressing technique (ODT) [17].
    Lesions with hypertrophic scar properties often improve when moisturizing heparinoid and silicone preparations are applied on their own.
    Silicone gels and creams are widely used globally to manage scars: it is believed that they mainly improve scars by moisturizing them [33, 36].
  Attention
    Corticosteroid ointments and creams may cause steroid acne [37] and capillary dilation if they come into contact with normal skin. Care should be taken when prescribing these preparations for long-term unmonitored use.
    If active acne lesions co-localize with keloids and hypertrophic scars, they may be exacerbated by corticosteroid ointment or cream therapy.
  Goal
    The goal of these treatments is to reduce inflammation, thereby ameliorating scar symptoms such as pain and itch and improving the color, elevation, and contracture of the scar. The ultimate aim is to promote scar maturation [3, 36].
4. Oral medicines (tranilast, Saireito) (Fig. 25)
  Concept
    Randomized clinical trials show that the antiallergy drug tranilast (Rizaben®) effectively improves the symptoms of keloids and hypertrophic scars [38–40].
    Tranilast ameliorates allergic reactions by suppressing mast cell activities. These activities also play an important role in pathological scarring because they involve the release of chemical mediators such as histamine. These mediators promote pathological scar growth by increasing fibroblast collagen production and vascular endothelial cell proliferation [41–44].
    A Chinese medicine called Saireito is thought to effectively reduce inflammation and has been shown to inhibit fibroblast proliferation [45, 46]. Thus, it may help to ameliorate pathological scars, although this possibility remains to be formally shown.
  Hints and tips
    While tranilast seems to have a weak effect on single small keloids and hypertrophic scars, it appears to be more effective on large burn-induced keloids and hypertrophic scars and in patients with large numbers of pathological scars. This may reflect the presence of systemic factors in these patients that promote pathological scarring and that can be alleviated by a systemically distributed oral medicine like tranilast.
    Nevertheless, it is unlikely that oral medicines on their own can significantly improve keloids and hypertrophic scar: this is because local proinflammatory factors such as skin tension have a particularly powerful effect on scar growth. Thus, tranilast and Saireito should be used in combination with external preparations.
  Attention
    Symptoms of bladder inflammation have been reported to be a side effect of tranilast [38]. Treatment with this medication should be discontinued if these symptoms occur. In addition, tranilast can cause liver injury. It is also contraindicated in pregnant women and women who may become pregnant.
    Interstitial pneumonia has been reported to be a side effect of Saireito [46].
  Goal
    The goal of these oral medicines is to improve subjective scar symptoms such as itching, pain, and redness.
5. Rest/fixation therapy (administered by applying fixation tape or gel sheets) (Fig. 26)
  Concept
    The growth of keloids and hypertrophic scars is promoted by skin tension that pulls on the scar [5, 11, 47]. Consequently, existing scars can be improved by applying fixation tape or gel sheets. It has been formally shown that gel sheets can reduce local skin tension on keloids and hypertrophic scars [47–49].
    Fixation tape and gel sheets can also promote scar maturation by moderately moisturizing the surface of the scar [33].
    A meta-analysis of 20 randomized controlled trials found overall that silicone gel sheets may be useful for preventing or treating keloids and hypertrophic scars. However, since the quality of the trials was poor, this finding should be interpreted very cautiously [51].
    Fixation tape is mainly made of paper or silicone. The gel sheets are made of silicone or polyethylene.
  Hints and tips
    It is not necessary to replace the fixation tape every day: this will help to prevent irritant contact dermatitis or epidermal damage due to tape replacement. Indeed, it is recommended to replace the tape only when it detaches naturally.
    If the patient feels scar itching, corticosteroid ointment or cream can be applied onto the fixation tape: the ointment/cream will penetrate the tape. However, long-term blind use of corticosteroids in this manner should be avoided because of the risk of side effects such as steroid acne [37].
    Gel sheets are generally removed and cleaned daily. They can be re-used after washing until the adhesive material has disappeared.
  Attention
    In summer, sweating may lead to an excessively moist environment under the fixation tape/gel sheet. If this environment is prolonged due to extensive tape/gel sheet use, it can lead to fungal infection of the scar. Therefore, it is essential that the scar is cleaned every day under the shower in summer.
    By contrast, winter can lead to an overly dry environment around the scar. Since fixation tapes and gel sheets have moisturizing properties, they should be used assiduously during winter to prevent proinflammatory drying of the scar.
    The fixation tape/gel sheet should be large enough to firmly cover the affected area, thereby protecting it from tension on the scar. This will also ensure that the fixation tape/gel sheet moisturizes the area sufficiently.
  Goal
    The goal of this treatment is to improve the objective symptoms of pathological scars, including their redness.
6. Compression therapy (administered by applying bandages, supporters, garments, etc.) (Fig. 27)
  Concept
    Compression therapy has long been used to treat hypertrophic scars that arise from burns [52, 53]. It is also widely used as a conservative treatment for keloids and hypertrophic scars in general [48, 49, 54–56].
    This therapy is believed to act by placing pressure on the blood vessels in and near the scar. This in turn reduces the blood flow in and to the scar, thereby decreasing the influx and local circulation of proinflammatory agents such as immune cells and cytokines [5].
  Hints and tips
    It is recommended to use supporters and knee braces on scar-affected limbs and joints, while corsets are suitable for abdominal scars and chin caps are appropriate for lower jaw scars. Bandages and garments are suitable for scars in other body areas.
  Attention
    The heat of summer may limit the continuous use of a compressive material. Therefore, in summer, it may be necessary to choose a compressive material that had good ventilation.
    Bandages and the gum of compression garments may induce itching. If itching arises, the use of these compressive materials should be stopped temporarily [49].
  Goal
    The goal of compression therapy is to improve objective scar symptoms such as redness.
    Compression therapy can also be used to prevent keloids and hypertrophic scars from arising after surgery. In this case, the therapeutic goal is to induce surgical scar maturation.
7. Surgical excision and closure with simple sutures (Fig. 28)
  Concept
    In many cases, closure after keloid/hypertrophic scar excision can be achieved with simple sutures. However, great care should be taken to avoid placing tension on the reticular dermis of the surgical wound because this tension will induce the chronic inflammation that will ignite pathological scar recurrence. There are several strategies that can limit tension on the surgical wound, as follows.
    The dermal sutures should be placed with minimal tension.
    The trunk area is subject to particularly strong skin tension. Therefore, when excising a pathological scar on the trunk, it is recommended to remove the fatty tissue under the scar and then to undermine the fascia. Thereafter, the fasciae should be sutured together. This will cause the tissues above the fasciae to approximate each other closely, thereby allowing the wound to be easily closed by first dermal sutures and then epidermal sutures with minimal tension [57–61] (Fig. 29).
    In the case of long post-excision wounds, it is advisable to divide the wound by using z-plasties: these will disperse the tension on the length of the wound [18, 61, 62].
  Hints and tips
    The dermis only recovers about 80% of its strength by the third month after surgery [63]. Therefore, it is advisable to perform subcutaneous suturing with a polydioxanone thread that can maintain its tensile strength for at least 3 months. Polyglactin thread is not suitable because it is more readily absorbed and thus maintains its tensile strength for a short period only.
    An absorbable thread that is coated with an antimicrobial agent can be used [64].
    If z-plasties will be added, it is best to design them after the wound edges have been closely approximated by fascial sutures.
  Attention
    When applying dermal sutures, be careful not to place the thread on the shallow part of the dermis because this can damage the hair roots. This in turn can induce folliculitis and epidermal cysts, which can trigger the growth of new keloids and hypertrophic scars.
    Since non-absorbable threads can cause foreign body granulomas or suture abscesses, it is best to use absorbable threads for subcutaneous and dermal suturing.
  Goal
    The goal of this surgical approach is to completely eliminate the pathological scar (along with its symptoms) without igniting recurrence or new pathological scars.
8. Surgical excision using the core excision method or partial resection (Fig. 30)
  Concept
    Sometimes the keloid or hypertrophic scar is so huge that it is not technically feasible to excise it entirely. Total excision will also not be suitable if it could cause significant deformity. In these cases, it is best to perform core excision or partial excision [65–67]. In core excision, the inner fibrous layer of the scar is excised and the defect is covered by a flap composed of the surface tissues of the scar. In partial resection, only part of a large lesion or only a few of multiple lesions are excised.
    When applying partial excision, it is important to subject the remaining lesions to postoperative treatments, specifically, radiotherapy and/or external corticosteroid treatment via injection and tape/plaster.
  Hints and tips
    The core excision method is particularly suitable for pathological scars in the cartilaginous part of the auricle. This is because total removal of these scars can lead to deformity [66, 67].
    When the lesion is huge and on highly tense body areas such as the anterior chest or shoulder, partial resection that only removes a strongly elevated area of the scar can be performed. Partial resection can also be used to excise an epidermal cyst with a complicated infection.
    In terms of the postoperative radiotherapy after partial excision, it has been suggested that the radiation dose should be equivalent that used in radiation monotherapy because its target is not only the excision wound but also the remaining lesions. Radiation monotherapy doses are higher than postoperative radiation doses (e.g., 37.5 Gy [81] vs. 30 Gy [76] for keloids) (see “Rationale behind each treatment for keloids and hypertrophic scars” section Nos. 11 and 12). However, it is possible that the total radiation dose can be reduced by concomitantly using corticosteroid tape/plaster treatment.
  Attention
    When performing the core excision method, be careful not to make the flap too thin because this could hamper the flow of blood to the wound edge.
    Partial resection has relatively poor cosmetic outcomes. Therefore, total resection followed by closure with primary sutures should be tried as much as possible.
  Goal
    The goals of this surgical approach are to remove pathological scars without inducing deformity and to remove problematic parts of the scarred area that are exhibiting strong inflammation or infection. Notably, removing the highly inflamed parts of the scarred area not only relieves some of the subjective symptoms, it can also help to ameliorate the remaining scars.
9. Surgical excision followed by z-plasty (Figs. 31 and 32)
  Concept
    The decision to use z-plasties when closing after pathological scar excision depends on the orientation of the incision line, the body site on which the scar is located, and how long the post-excision wound is, as follows.
    If the incision line follows the direction of predominant skin tension, it is recommended to perform one or more z-plasties to disperse the tension that will otherwise pull on the length of the scar [18, 61, 62].
    If the scar is on very tense areas such as the anterior chest, z-plasties are highly recommended.
    If the wound is more than 10 cm long, z-plasties are recommended even if the wound line lies perpendicular to the direction of predominant skin tension.
    If the lesion has strong keloid-like characteristics (JSS 2015 score of 16 points or more), it is important to perform postoperative radiotherapy after excision and z-plasty. This is because if the keloid recurs, it is likely to be larger and longer than the original keloid [55, 65].
  Hints and tips
    A 60° z-plasty lengthens the wound. If such wound lengthening will be a problem esthetically, it is recommended to perform z-plasties that have an acute angle (e.g., 45°). However, this approach should be applied with care because triangular flaps with an acute angle may suffer from blood flow problems.
    When transposing and suturing the triangular flaps of a z-plasty, the triangular flaps should not be pulled into place manually and then closed with dermal sutures: this will place great tension on the dermis. Instead, subcutaneous sutures should be placed under the flaps so that the triangular flaps transpose themselves naturally [18, 61].
  Attention
    If the triangular flap of the z-plasty is too large, it will lead to a wide wound. This may cause a problem esthetically. Consequently, one side of the triangular flap should be approximately 1 cm or less.
  Goal
    The goal of this surgical approach is to remove the pathological scar and its subjective symptoms while preventing its recurrence.
10. Surgical excision followed by reconstruction with skin grafts or flaps (Fig. 33)
  Concept
    If excising the keloid or hypertrophic scar leads to a wound that cannot be closed by low tension primary sutures, it should be reconstructed with skin grafts or flaps [68, 69]. However, in this case, it is particularly important to apply postoperative therapies that prevent recurrence, particularly radiotherapy.
    It is also important to apply the same postoperative recurrence prevention methods to the skin graft or flap donor site [70–73].
  Hints and tips
    Local skin flaps can be classified as skin-pedicled and island flaps. If possible, the skin-pedicled flaps should be chosen over island flaps because the skin pedicle stretches after surgery, thereby efficiently releasing the tension on the wound/scar edges. Island flaps do not benefit from such stretching because they are completely surrounded by stiff scar tissue [74].
    Skin flaps relieve postoperative contractures better than skin grafts.
  Attention
    If the pathological scar recurs after excision and reconstruction with skin grafts or island flaps, the recurrence will arise from the edges of the scars. To prevent these large and unsightly defects from forming, it is recommended to subject the margin of the skin graft or flap to postoperative radiation.
    For skin grafts, radiotherapy should be performed 1 week after surgery (if the graft has survived).
    For skin flaps, radiotherapy may be performed starting the day after surgery if the flap does not have blood flow defects. However, if the flap exhibits congestion or ischemia, radiotherapy should not be performed until the blood flow in the flap has stabilized.
  Goal
    The goal of this surgical approach is to eliminate large pathological scars and their symptoms and to close the resulting large wound without inducing recurrence.
11. Postoperative radiotherapy (Fig. 34)
  Concept
    Radiotherapy strongly inhibits inflammation, perhaps by impairing immune cell function and the formation of neovasculature. Extensive evidence suggests that postoperative radiotherapy significantly reduces the risk of recurrence after pathological scar excision. For example, if radiation treatment with a biologically effective dose (BED) of at least 30 Gy* is completed within 1 week after keloid resection, the keloid recurrence rate improves to 10% or less (compared to a rate of 50–80% after surgery alone) [76, 77]. * BED is calculated as 1 time dose × number of irradiations × [1 + 1 time dose / (α/β ratio)]. In previous reports [76, 77], the α/β ratio of keloid tissue was estimated to be 10 Gy. Thus, a 30 Gy BED for keloids can be achieved with a regimen of 20 Gy/4 fractions/4 days or a regimen of 18 Gy/3 fractions/3 days.
    The most common postoperative radiotherapy regimen for keloids is 15 Gy/3 fractions/3 days [75].
    Regarding the start of radiation, many papers state that radiotherapy should be performed immediately after the operation. However, there are also some studies that have found that delaying the start of radiation does not affect the recurrence rate. Whether delaying the initiation of radiotherapy could actually improve the non-relapse rate has not yet been studied.
    The 2016 edition of the Japanese guideline to radiotherapy planning for benign diseases [75] does not state that standard treatment regimens can induce secondary carcinogenesis. Nevertheless, it is recommended that the patient should be informed of the possibility of secondary carcinogenesis. The treatment should only be administered if the patient then consents.
    If an electron beam is used to irradiate an operative wound, the irradiation field should include a margin of 5 to 10 mm. This is because the radiation dose drops at the edge of the irradiation field [75].
  Hints and tips
    When the BED at an α/β ratio of 10 Gy exceeds 30 Gy, the non-relapse rate approaches a plateau. Therefore, it is not recommended to increase the dose beyond 30 Gy because there will be little added clinical benefit and more side effects.
    Generally, the recurrence rates of resected keloids on the anterior chest, scapula, and upper pubic region are high. By contrast, the recurrence rate of resected earlobe keloids is low. Therefore, site-specific radiation protocols are recommended [75, 78]. Thus, it is currently recommended that the anterior chest, scapula, and upper pubic region receive 20 Gy/4 fractions/4 days while the earlobe receives 10 Gy/2 fractions/2 days and other areas receive 15 Gy/3 fractions/3 days (see “Site-specific treatment protocols” section).
    Electron beam is widely used for radiotherapy after pathological scar resection. There are also reports of intra-tissue irradiation and mold irradiation using brachytherapy [79, 80].
  Attention
    Increasing the fraction dose and the total dose is likely to elevate the risk of side effects such as pigmentation [75].
    Areas where the thyroid or mammary gland lies directly under the skin should not be subjected to radiotherapy because these organs have a high risk of developing cancer [77]. Instead, excision wounds on these areas should be treated postoperatively with another therapy such as corticosteroid tape/plaster or injection. However, given that radiation sensitivity varies with age and is low in elderly patients, there is room for considering radiotherapy on these areas in elderly patients.
    Radiotherapy should not be given to children because they are in a radiosensitive growth stage [77]. If surgery is required in these patients, an alternative combination therapy such as corticosteroids tape/plaster or injection treatment should be considered.
  Goal
    Postoperative radiotherapy aims to control recurrence after pathological scar resection.
12. Radiation monotherapy (Fig. 35)
  Concept
    The evidence for the ability of radiation monotherapy to treat keloids remains relatively poor. Consequently, it is currently recommended to treat pathological scars with surgery combined with postoperative radiotherapy [80]. However, radiation monotherapy can be used to improve pain and itch in the very few cases in which surgery is difficult to perform.
  Hints and tips
    It is thought that radiation monotherapy with 24–30 Gy/4–5 fractions/2–5 weeks can have good results with keloids and hypertrophic scars. One retrospective cohort study [81] also showed that radiation monotherapy of 64 patients with bulky unresectable keloids with 37.5 Gy/5 fractions/5 weeks induced significant regression in 97% at 18 months.
  Attention
    Radiation monotherapy is not recommended for young people because the total dose is higher than that provided by postoperative radiation: this increases concerns regarding the risk of secondary carcinogenesis.
  Goal
    Radiation monotherapy aims to suppress the inflammation in pathological scars and to induce scar maturation.
13. Laser therapy (Fig. 36)
  Concept
    Laser therapy is thought to be effective for hypertrophic scars and keloids because heightened vascular proliferation plays a key role in pathological scar formation and progression. Since vascular lasers disrupt the high blood flow in the scars, they decrease fibroblast proliferation, type III collagen deposition, and histamine release. The vascular lasers that can achieve this are the pulsed dye laser (585 nm [82–84] or 595 nm [85]) and the YAG laser (532 nm [83, 85] or 1064 nm [86, 87]). The main clinical effect of vascular laser therapy is to decrease erythema and pruritus [84, 87–91]. Flat keloids/hypertrophic scars are particularly suitable for laser therapy because the laser beam can fully reach the blood vessels in these scars.
    Fractional resurfacing is a concept of cutaneous remodeling in which a laser generates zones of microthermal injury that are surrounded by normal untreated tissue. This fractional laser therapy induces a wound healing response that involves heat shock proteins and myofibroblasts and leads to increased collagen III production. This in turn promotes scar remodeling. This therapy is suitable for pathological scars: a randomized controlled study showed that the keloids and hypertrophic scars of 30 patients responded significantly to fractional laser therapy [ref].
    Fully ablative laser therapy is not recommended for pathological scars because it associates with high recurrence rates [92, 93].
    Keloid therapy with high/low response level laser therapy (HLLT/LLLT) has also been reported. However, the results may vary depending on which device is used [94].
  Hints and tips
    The 595-nm pulsed dye laser penetrates deeper than the 585-nm pulsed dye laser. The 585–595-nm pulsed dye laser protocols that were commonly used in the past recommended an energy setting between 3 and 10 J/cm^2^ and a pulse duration of 0.45–10 ms when using a 7 or 10 mm spot. The treatments were performed 2–4 times with intervals of approximately 4–8 weeks [95].
    Ablative fractional lasers can cause thermal injury at deeper levels than the non-ablative fractional lasers: therefore, ablative fractional lasers are more effective for thicker scars.
    A combination treatment composed of vascular laser and fractional laser therapy is more effective for pathological scars than monotherapy with either laser.
    During laser therapy, it is advisable to hold the laser light beam perpendicular to the scar surface: this 90° orientation should be maintained as the beam is passed over the curvature of the scar surface. When irradiating the boundary of the scar, it is permissible to irradiate some of the adjacent normal skin as well (Fig. 37)
  Attention
    It is recommended to conduct laser therapy while cooling the skin to protect the skin’s surface.
    Pulsed dye laser and YAG laser have only limited efficacy with thick keloids and hypertrophic scars [87, 95].
    Combining laser therapy with corticosteroid injection can improve the outcomes of laser therapy. However, it is not advisable to perform pulsed dye laser therapy immediately after steroid injection because of the lack of target chromophore.
  Goal
    The goal of laser therapy is to improve the texture and color of the skin and to decrease scar height, hyperpigmentation, erythema, and pruritus.
14. Make-up therapy (Fig. 38)
  Concept
    Special medical make-up techniques such as “Rehabilitation Make-up®” can temporarily improve the appearance of keloids, hypertrophic scars, and mature scars [56, 96–98].
    Once the patients learn the technique, they can make themselves up when going out.
    Once the patients realize that they can temporarily improve their appearance themselves, their mental health improve, perhaps because it allows them to accept the appearance of their scars. This may also yield more positive attitudes to the treatment of their scars [56, 98].
  Hints and tips
    It is difficult to improve the appearance of highly elevated keloids, hypertrophic scars, and mature scars with make-up therapy. Therefore, it may be best to reduce the thickness of such scars with steroid injection or plaster before commencing the make-up therapy.
    If the scar is slightly rough, it can be covered by thin tapes, after which the foundation is placed onto the tapes.
  Attention
    Make-up therapy cannot improve the inflammation that drives keloid and hypertrophic scar growth. Consequently, make-up therapy should be used as an adjunct to therapies that actively suppress scar inflammation.
  Goal
    The goal of make-up therapy is to provide patients with the confidence that they can hide their scars whenever they want.
15. Psychosocial health care
  Concept
    Patients with a scar on their face are highly likely to feel depressed and anxious about their appearance. This is particularly true for girls and women and patients who suffer (or suffered in the past) from mental ill-health [99].
    Children who develop scars on their body after receiving burn injuries are also likely to develop mental health problems [100, 101].
    Psychosocial care is helpful for improving the quality of life of patients with scars.
  Hints and tips
    Burn patients, especially pediatric patients, should be supported by the mental health acute care team and the psychological and social care team in the hospital before discharge [102]. After discharge, the patients should be aided by the municipal children care support group and volunteer organizations run by the social welfare council [102].
    Make-up therapy not only improves patient satisfaction with their appearance, it can also decrease the anthrophobia that these patients sometimes develop [98].
  Attention
    The family of the patient may also need psychosocial health care.
    Scar treatment does not always improve the mental health of the patient. Some patients feel traumatized by the events that created the scars, even when their scars improve. In such cases, specialist psychological help is needed.
  Goal
    The goal of psychosocial health care is to help the patients to accept their altered appearance, to deal with the trauma caused by the scar-inducing event, and to engage fully in normal daily life.
16. Other treatments

*Cryotherapy*

- Concept
  - While burns readily induce keloids and hypertrophic scars, frostbite does not. It is believed that this reflects the fact that burn injuries increase the blood flow and inflammation in the wounded skin, whereas frostbite does not. This suggests in turn that cryotherapy with liquid nitrogen may be useful for keloids and hypertrophic scars.
  - In Japan, cryotherapy with liquid nitrogen was once widely employed to treat pathological scars. However, because it appeared to be only weakly effective, this technique eventually fell out of favor [103].
  - However, in recent years, multiple studies have shown that intralesional cryotherapy effectively reduces keloid volume, pain, and itch [104–109]. Nevertheless, it was also noted that the ulcers induced by intralesional cryotherapy take a long time to heal. In addition, a significant side effect of this therapy is depigmentation [104–109].
  - Finally, it is difficult to completely remove pathological scars with cryotherapy and recurrence is common.

*5-Fluorouracil (5-FU) injection*

- Concept
  - 5-FU is an antineoplastic drug that effectively induces keloid flattening and is thus widely used for these pathological scars.
  - While the mechanism by which 5-FU improves pathological scars remains poorly understood, there is some evidence that it may inhibit fibroblast growth and TGF-beta-induced collagen type I expression.
  - 5-FU is widely administered via an intralesional injection, either on its own [110] or combined with steroid injections [111] or laser therapy [112, 113]. It has also been used after surgery [111].
  - One of the 5-FU monotherapy regimens that has been reported to be relatively effective consists of low-dose 5-FU (less than 5 ml of a 2–4 mg/ml preparation) that is injected into the lesion once every 2 weeks [114].
  - A systematic review suggests that the clinical effectiveness of 5-FU injections is unstable since recurrence rates of up to 47% have been observed [115].

*Botulinum toxin (BTX) injection*

- Concept
  - Several studies have reported using intralesional BTX injection to treat keloids or hypertrophic scars [116–118]. However, the effectiveness of this therapy and the mechanism by which it improves pathological lesions remain unclear.

*Autologous fat grafting*

- Concept
  - A recent prospective study showed that injecting hypertrophic scars and mature scars with autologous fat improves scar pliability [119]. However, the ability of this approach to improve other clinical scar variables is unclear. The mechanism by which this method improved scar pliability is also not known.
  - Moreover, a randomized clinical trial showed that when mature pediatric burn scars were injected with autologous fat, the treatment did not influence any scar variables, including pigmentation, vascularity, height, or pliability [120]. Studies on the effectiveness of this method in Asian people with scars do not seem to have been reported.

### Site-specific treatment protocols

**Fig. 39:**
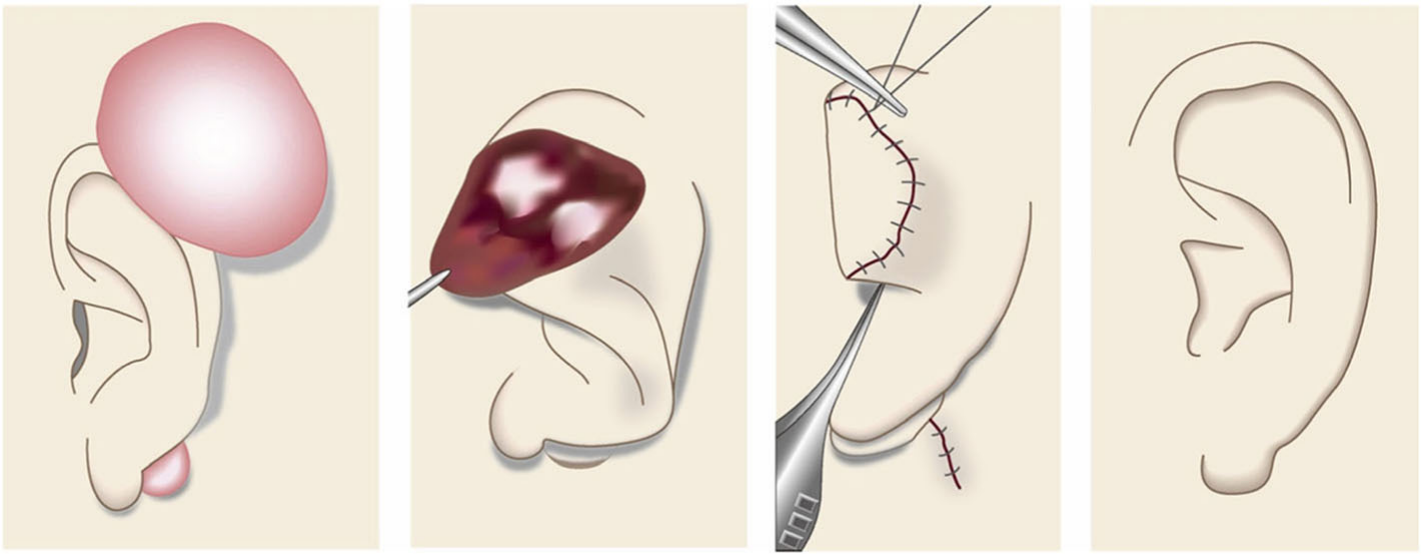
The core excision method for the cartilaginous part of the auricle. When excising keloids or hypertrophic scars from the cartilaginous part of the auricle, it is important to maintain the shape of the auricle. The core excision method is particularly suitable for this purpose

**Fig. 40:**
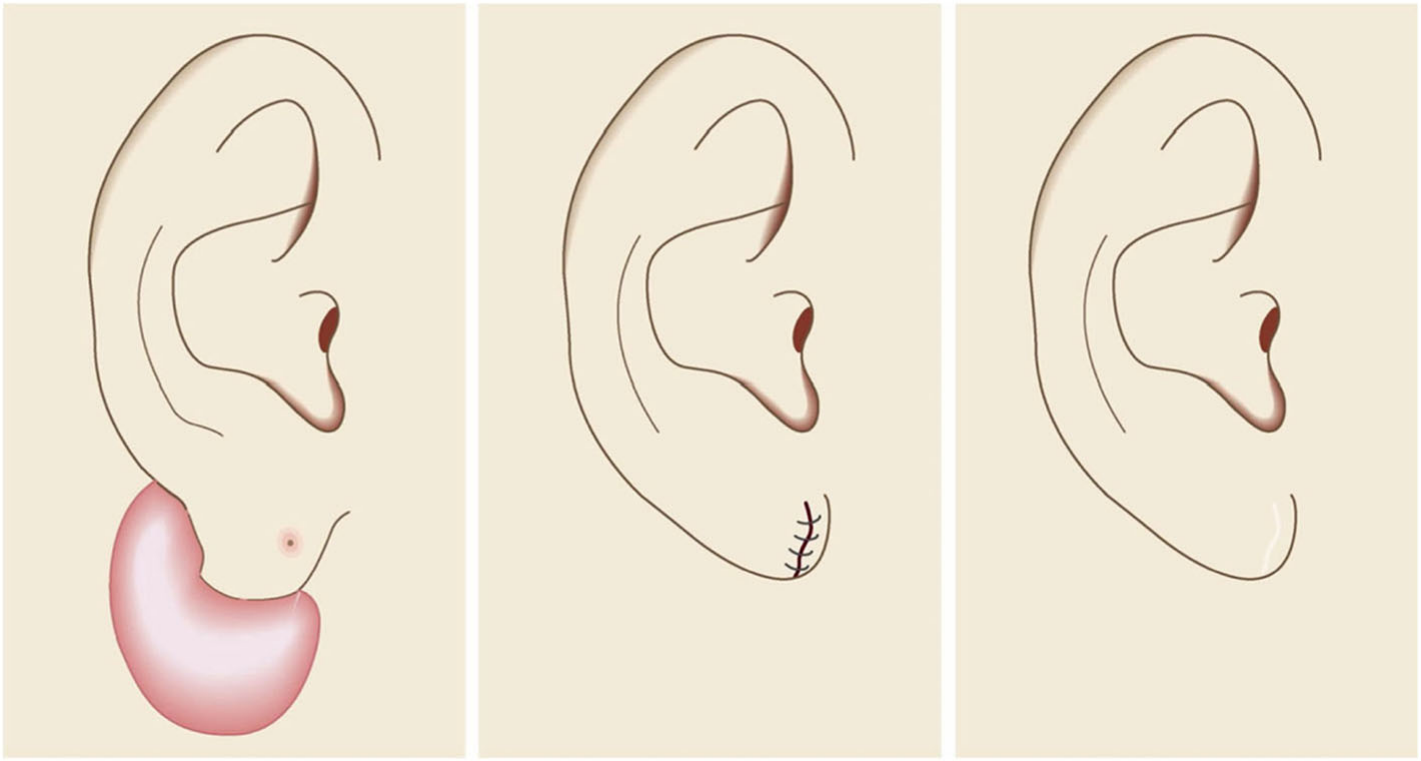
The wedge excision method for the earlobe. Many keloids and hypertrophic scars of the earlobe originate from the piercing hole. Most primary cases can be treated by wedge excision and simple suture, which maintains the shape of the ear lobe

**Fig. 41:**
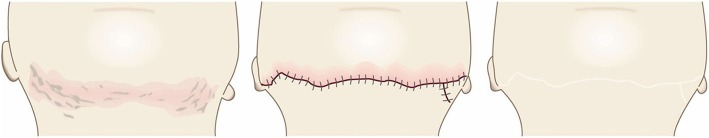
Simple closure method for the lower jaw. Multiple keloids and hypertrophic scars on the lower jaw can be converted into linear mature scars by surgery and postoperative therapies such as radiotherapy and/or corticosteroid tape/plaster and injection therapy

**Fig. 42:**
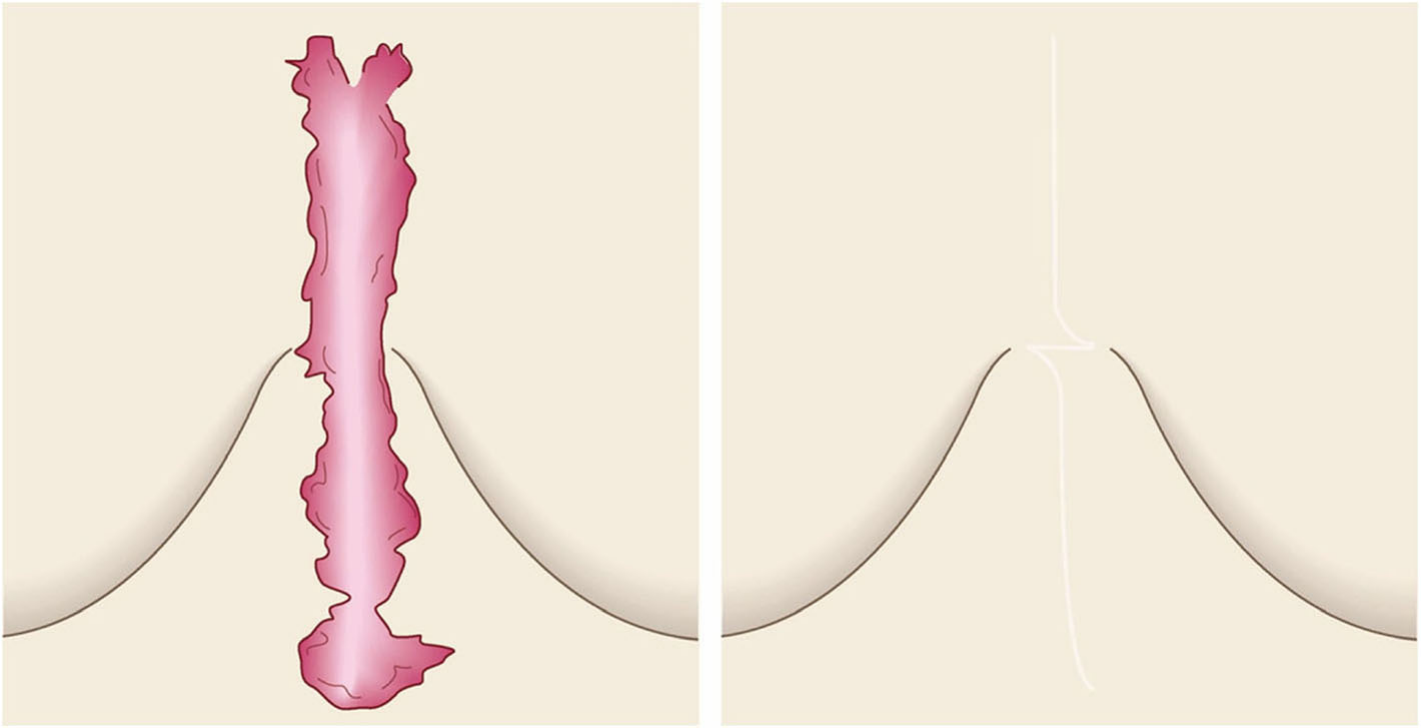
Simple closure method for the anterior chest wall (the scars developed from a midline chest incision). Surgery and postoperative radiotherapy is indicated for a broadening keloid/hypertrophic scar that is growing from a midline chest incision

**Fig. 43:**
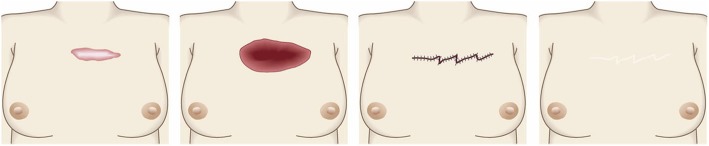
Z-plasties for the anterior chest wall (the scars developed from non-midline incisions or acne/folliculitis). Most of the keloids and hypertrophic scars that spread laterally on the anterior chest are due to acne/folliculitis and minor surgery. It is recommended to use z-plasty after excising these scars because this will disperse the horizontal skin tension on the wound

**Fig. 44:**
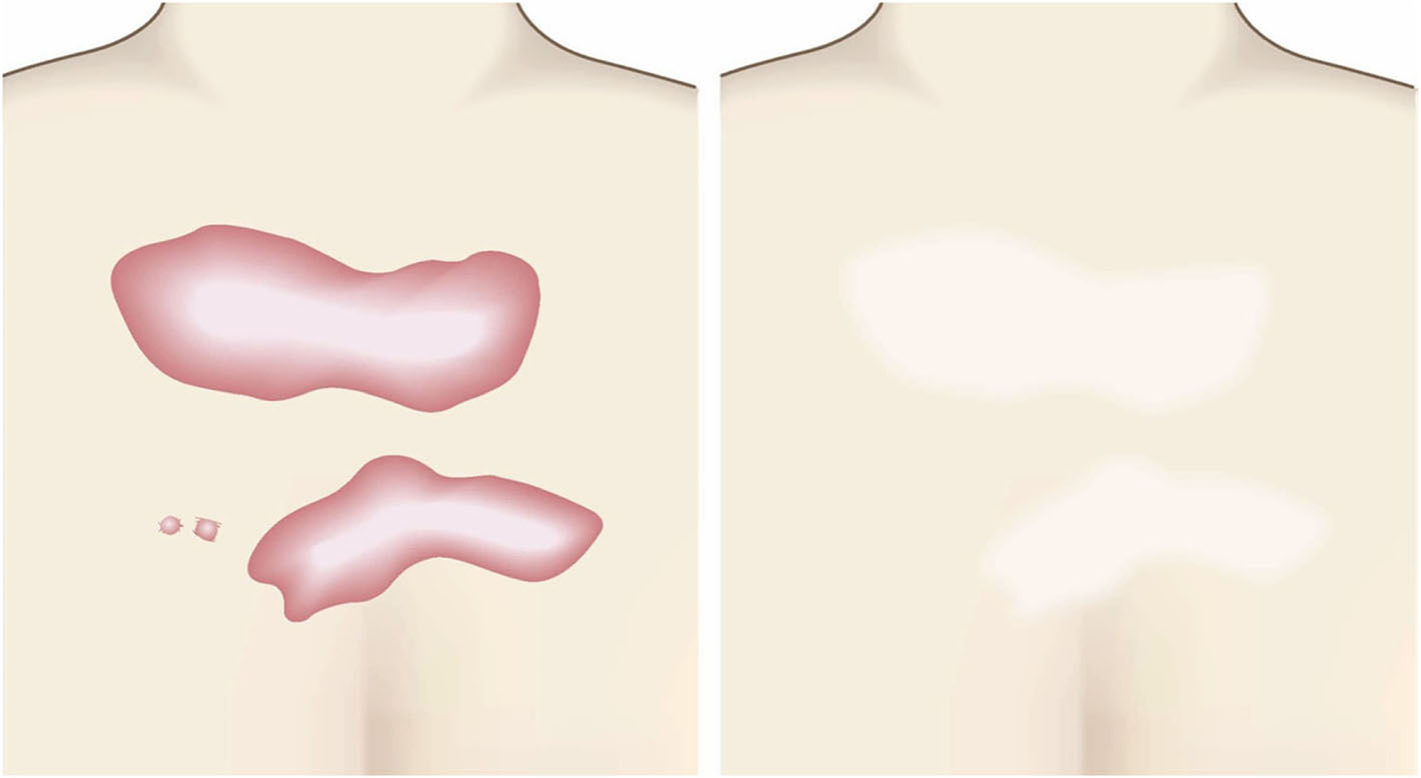
Conservative therapies for the anterior chest wall. Conservative therapies such as laser therapy will help keloids and hypertrophic scars on the anterior chest to mature. The scar shape will remain but it will be inconspicuous after the scar becomes mature

**Fig. 45:**
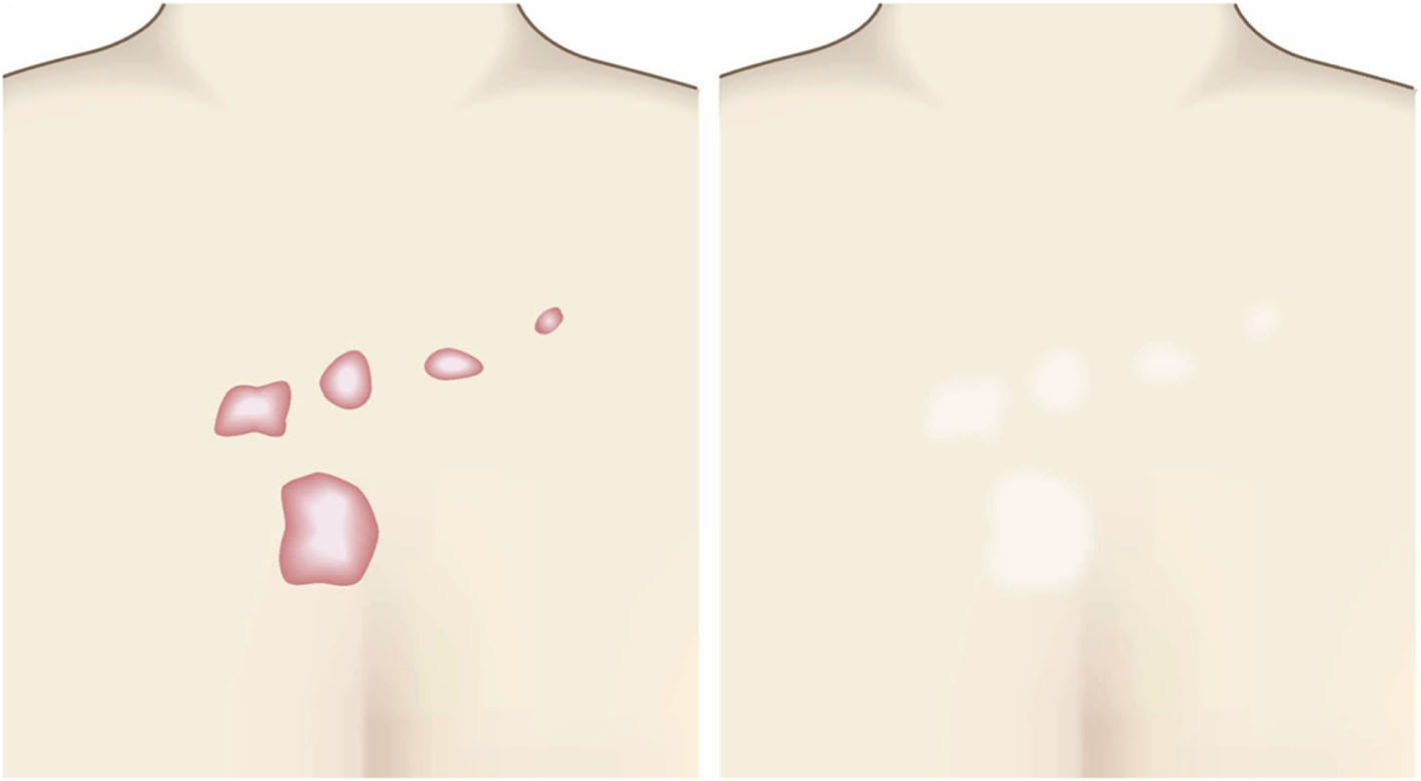
Laser therapy for the anterior chest wall. Conservative therapies such as laser therapy are particularly indicated for small keloids and hypertrophic scars on the anterior chest

**Fig. 46:**
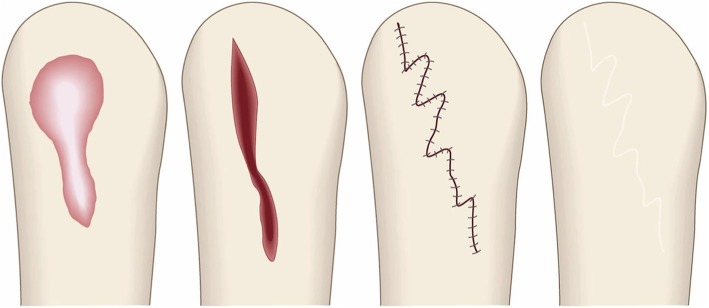
Z-plasties for the upper arm. After excising a keloid or hypertrophic scar on the upper arm, it is recommended add z-plasty to disperse the skin tension on the wound

**Fig. 47:**
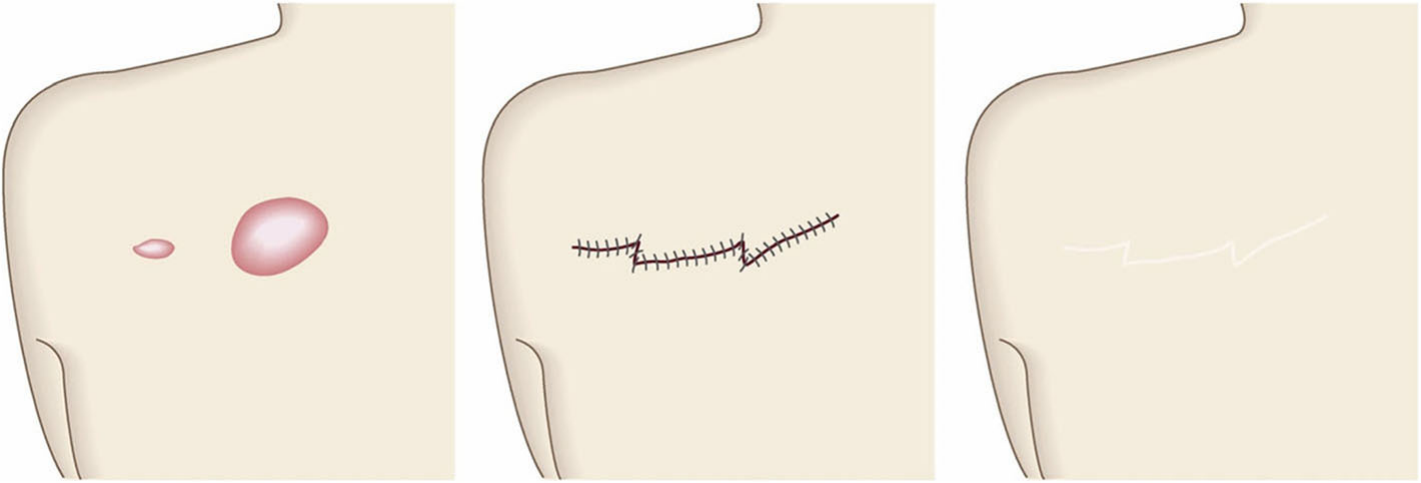
Z-plasties for the scapular area. If the wound left after excising a scapular keloid/hypertrophic scar is long, it should be closed with z-plasty to disperse the skin tension on the wound

**Fig. 48:**
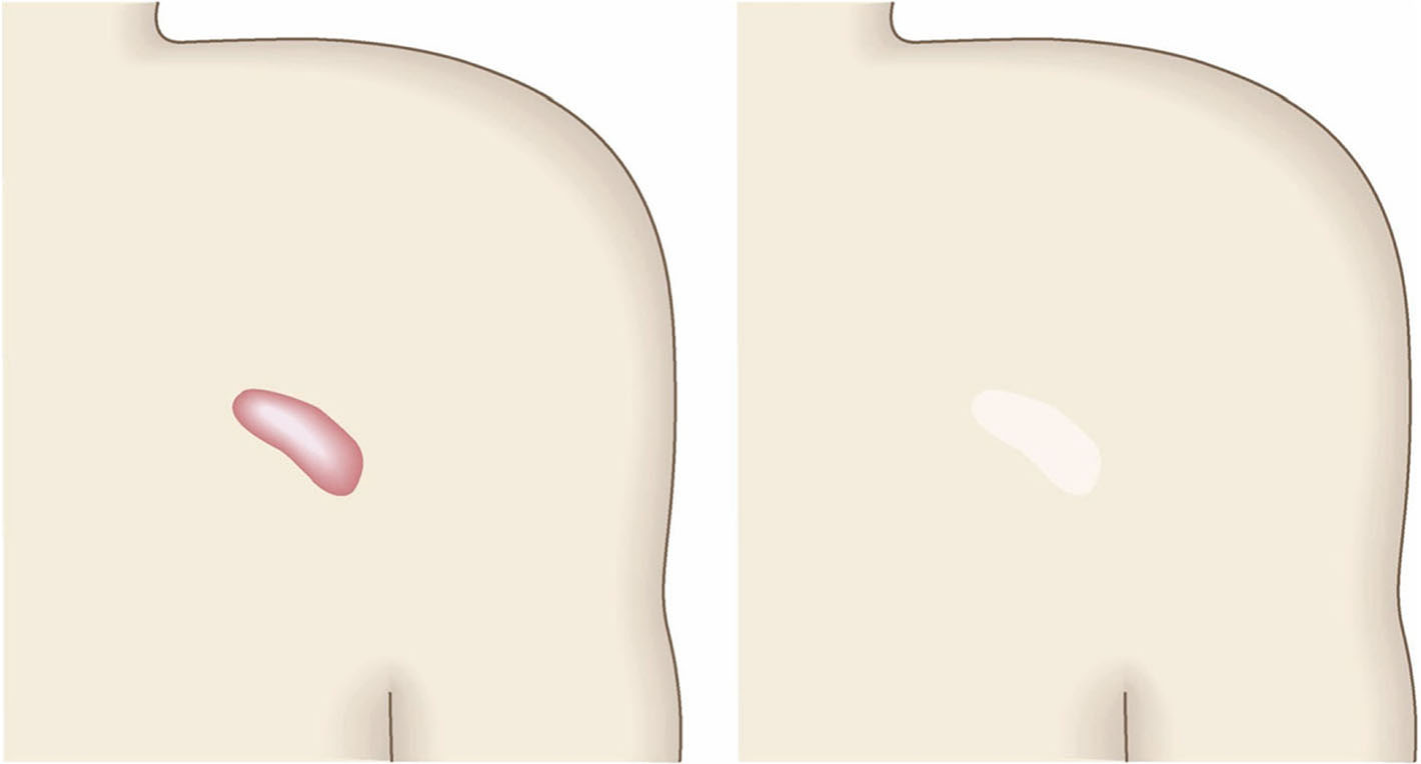
Conservative therapies for the scapular area. It is recommended to treat small keloids and hypertrophic scars on the scapula with corticosteroid tape/plaster or injection therapy

**Fig. 49:**
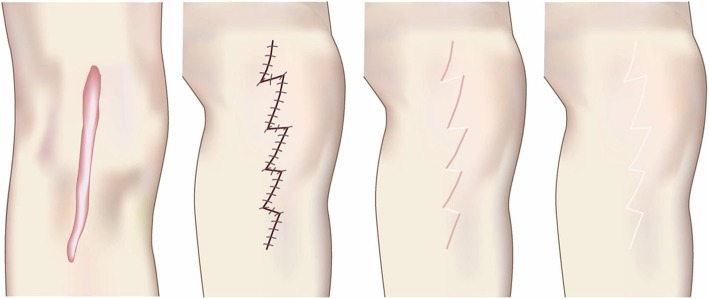
Z-plasties for joint areas (the hand, elbow, knee, and foot). If the pathological scar on a joint is thin and runs in the direction in which the joint is extended, it is recommended to completely excise the scar and perform z-plasties to disperse the tension on the wound

**Fig. 50:**
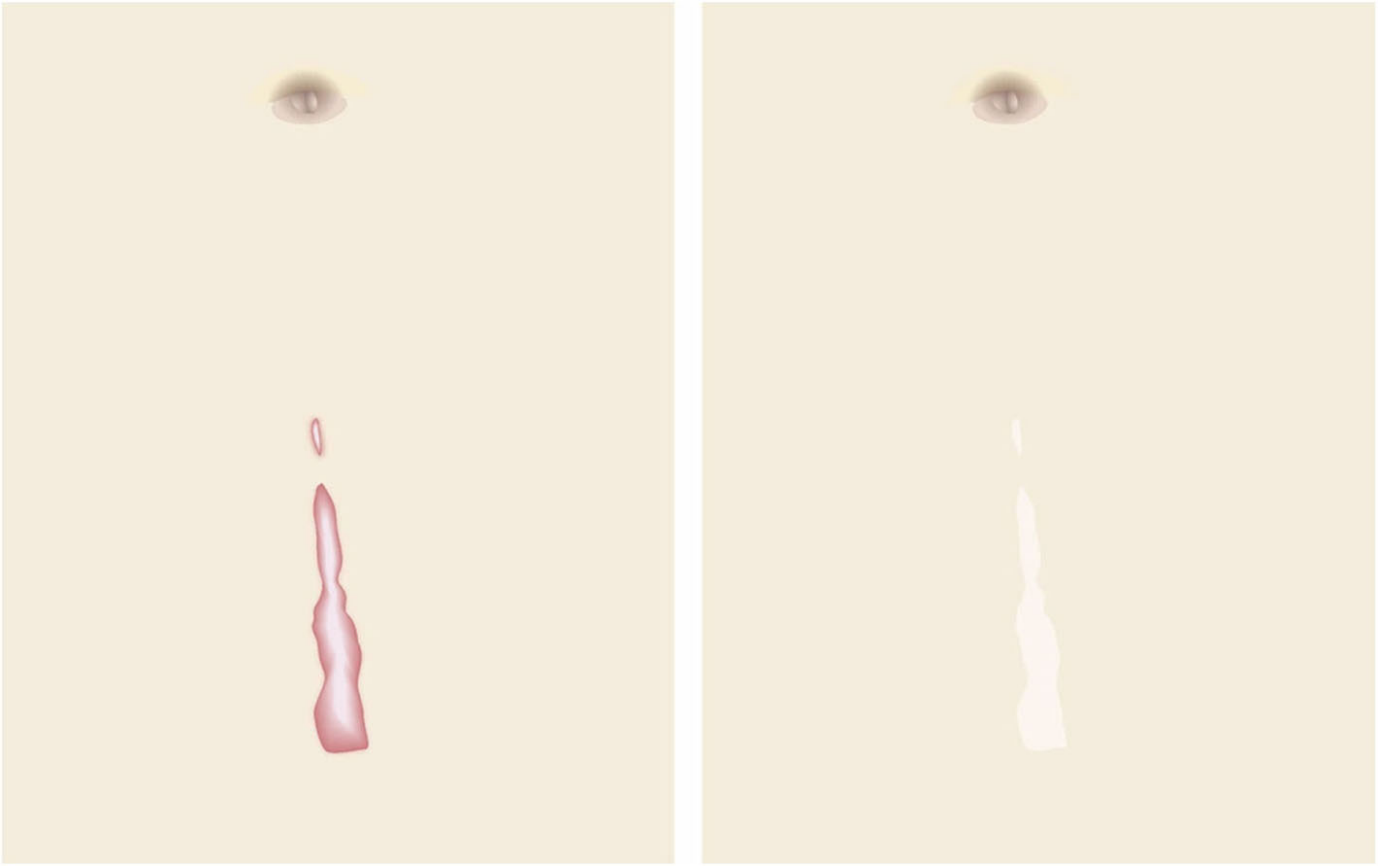
Conservative therapies for the abdomen. Keloids and hypertrophic scars that arise from a midline abdominal incision can be treated with conservative therapies. However, the shape of the scar will remain

**Fig. 51:**
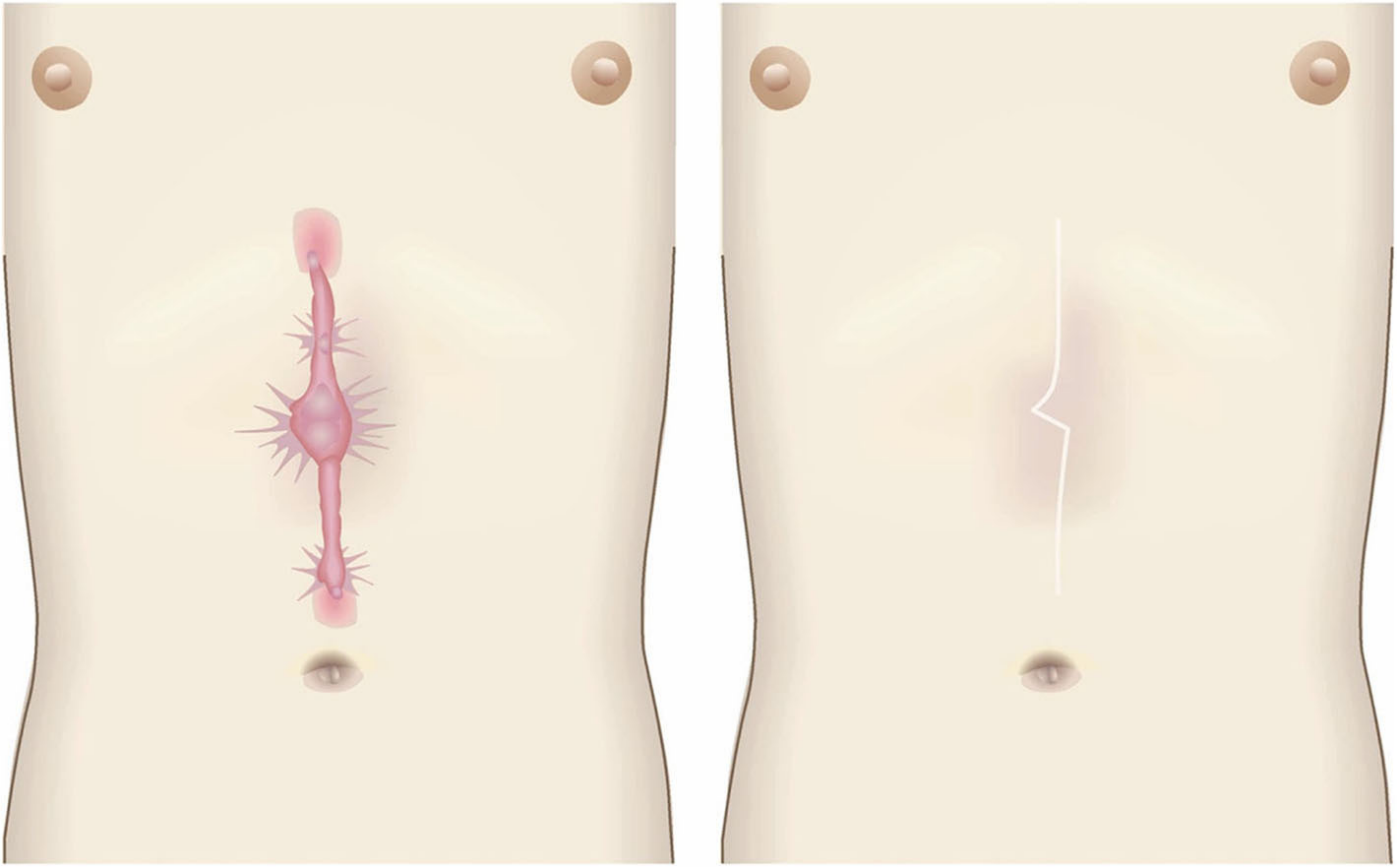
Z-plasties for the abdomen (the scars developed from an abdominal midline incision). If surgery is selected to treat a midline abdominal scar, it will yield a thin linear scar that is barely visible after maturation. If the scar is long, z-plasties should be applied to disperse the skin tension on the wound

**Fig. 52:**
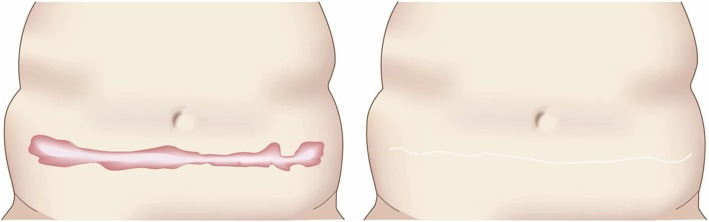
Simple suture method for the abdomen (the scars developed from non-midline incisions). Non-midline abdominal scars can often be removed completely and sutured primarily because the abdomen has a relatively large amount of skin

**Fig. 53:**
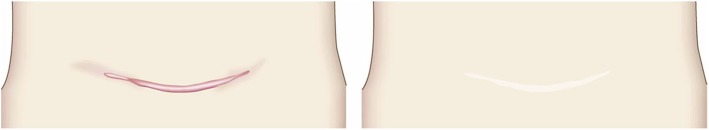
Conservative therapies for the abdomen (the scars developed from non-midline incisions). Conservative therapies such as laser therapy may also be suitable for non-midline abdominal scars

**Fig. 54:**
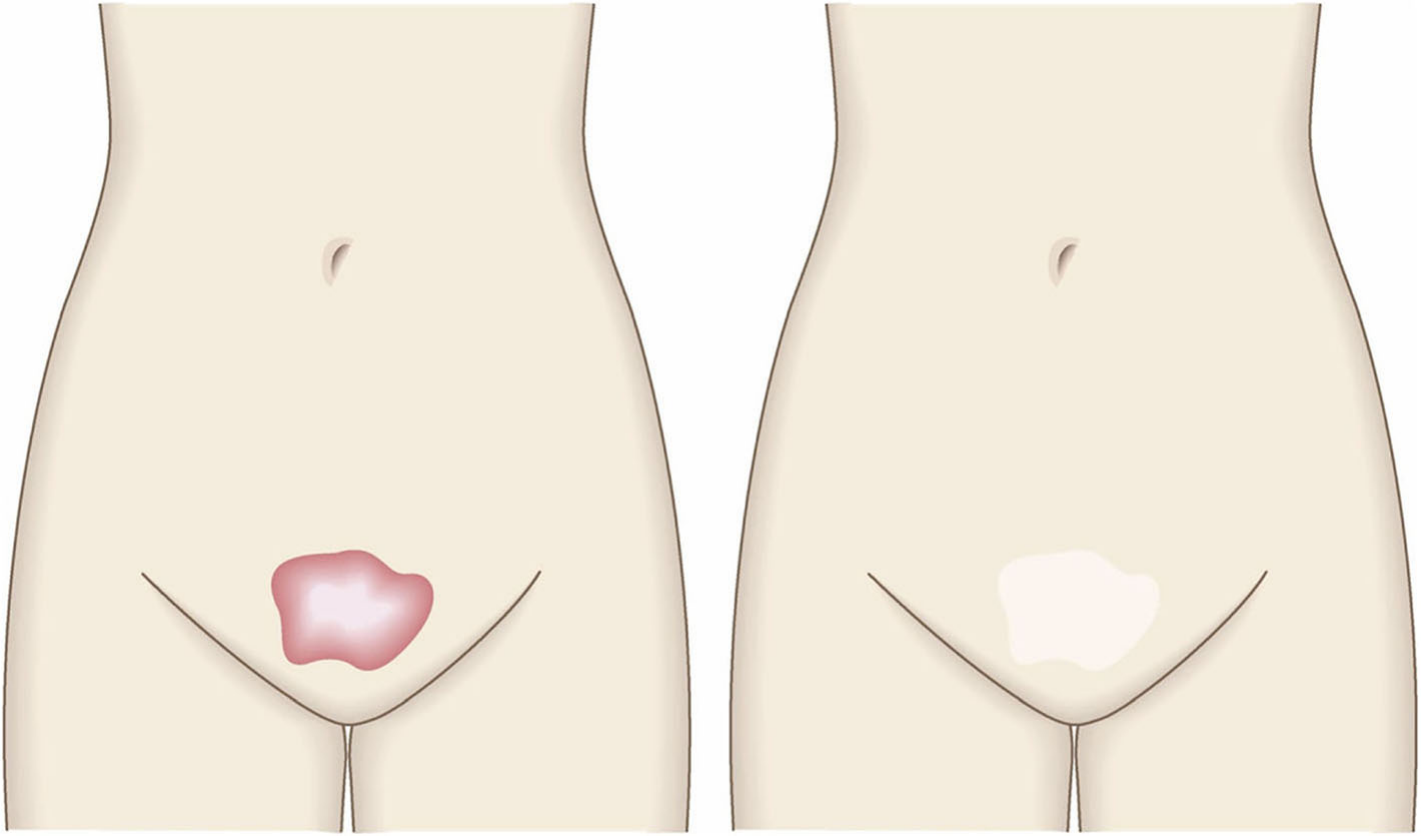
Conservative therapies for the suprapubic area. If a keloid or hypertrophic scar on the suprapubic region is infected, surgery is the first choice of therapy

**Fig. 55:**
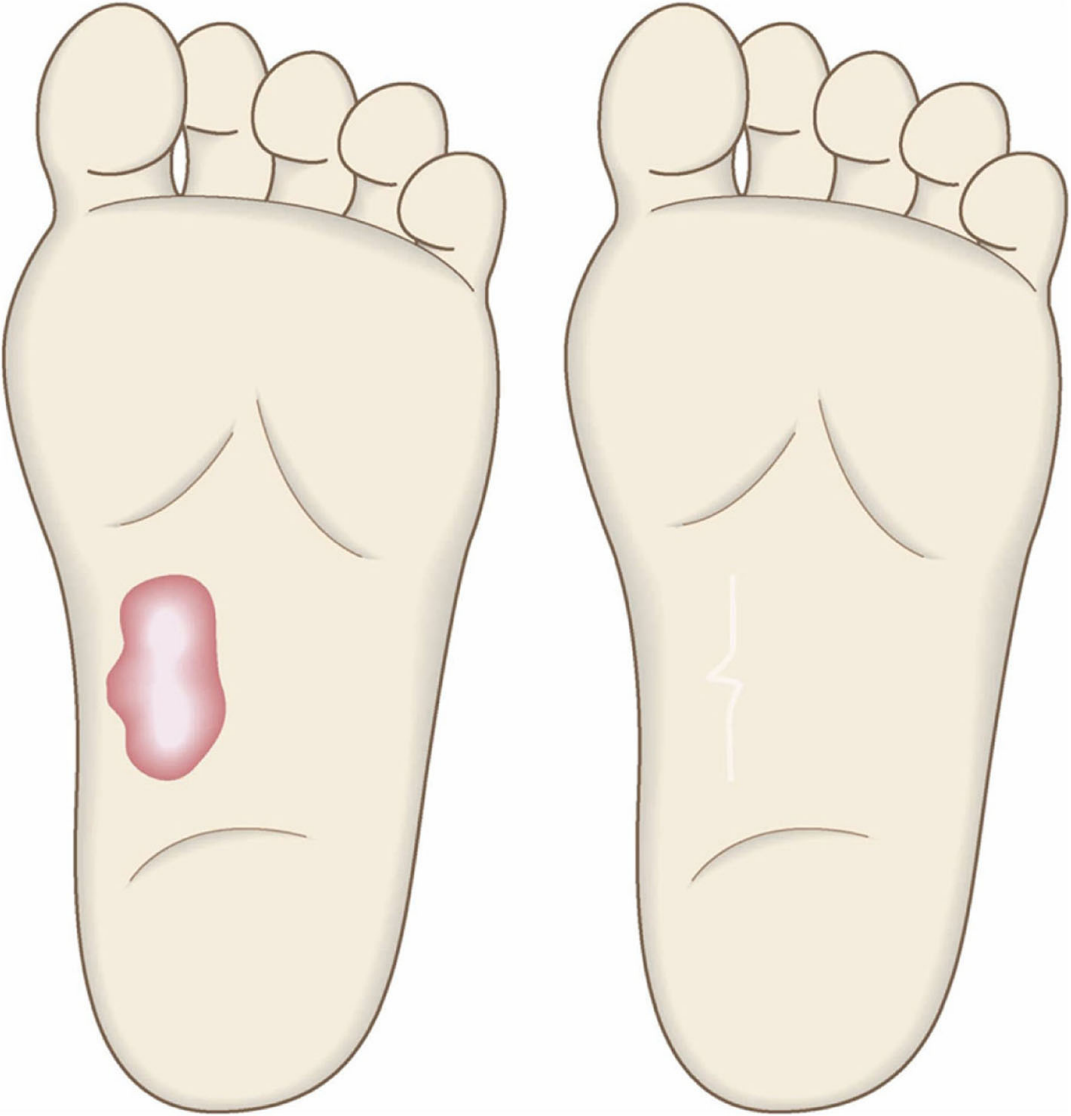
Other body areas. Although rare, keloids and hypertrophic scars can occur on the face, external genitals, and foot soles. Depending on the affected body region and lesion severity, conservative therapies and/or surgery followed by the appropriate radiotherapy protocol are recommended

1. Cartilaginous part of the auricle (Fig. 39)
  Small lesions on the auricular cartilage that have strong hypertrophic scar properties (JSS 2015 score of 6–15 points [2]) can be treated with corticosteroid tape/plaster or injections.
  Large or multiple auricular cartilage scars that have strong hypertrophic scar properties and all auricular cartilage scars that have strong keloid-like properties (JSS 2015 score of 16 or more points [2]) can be treated by surgery.
  If surgery is selected, it should be followed with postoperative radiotherapy or combined with corticosteroid tape/plaster or injection therapy [70–74, 76–78].
  During surgery, it is important to maintain the shape of the auricle: consequently, it is best to employ the core excision method [66, 67].
  While the ear piercings that generate pathological auricular cartilage scars often penetrate the cartilage, the pathological scars rarely involve the perichondrium: consequently, the perichondrium can be preserved during surgery.
  If the lesion is only elevated on the front or back of the auricle, it may be possible to cut out the elevation on one side, perhaps by spindle-shaped excision.
  Small auricular cartilage lesions can be resected by using wedge excision. However, in this case, a small z-plasty on the lateral side of the auricle should be considered because it will generate a smooth surface and thereby prevent scar contracture.
  Superficial sutures are sufficient for closing after auricular cartilage scar excision. A 6-0 nylon or polypropylene thread is recommended.
  The recommended postoperative radiotherapy protocol is 15 Gy/3 fractions/3 days [77, 78].
  It is recommended to apply wound fixation materials such as tape for 3–6 months after surgery.
  If the resected area exhibits signs of recurrence (e.g., scar induration, swelling, pain, and/or itch), corticosteroid tape/plaster [17–19] can often dampen the inflammation.
2. Earlobe (Fig. 40)
  Many earlobe keloids develop from the holes made for wearing earrings. However, some also arise from atheromas (epidermal cysts).
  Some benign and malignant tumors resemble keloids and hypertrophic scars on the ear lobe: an example is pseudolymphoma (Fig. 9). Consequently, differential diagnosis should be conducted carefully.
  Small earlobe lesions that have strong hypertrophic scar properties (JSS 2015 score of 6–15 points [2]) can be treated with corticosteroid tape/plaster or injections.
  The first choice for larger or multiple hypertrophic scar-type lesions on the earlobe is surgery. This is also true for all earlobe lesions that have strong keloid characteristics (JSS 2015 score of 16 or more points [2]).
  If surgery is selected, it should be followed by postoperative radiotherapy or combined with corticosteroid tape/plaster or injection therapy [70–74, 76–78].
  During surgery, if the pierced hole is in the middle of the earlobe, it is often possible to perform wedge excision and suturing [121, 122].
  When keloids and hypertrophic scars on the earlobe that have undergone surgical resection recur, they often adhere to the buccal skin. Consequently, it will be necessary to reconstruct the shape of the earlobe by performing z-plasty and/or applying local flaps [121, 122].
  Superficial sutures are sufficient for closing the wounds left by earlobe lesion excision. A 6-0 nylon or polypropylene sutures should be used.
  The recommended postoperative radiotherapy protocol is 10 Gy/2 fractions for 2 days [77, 78].
  It is recommended to apply wound fixation materials such as tape for 3–6 months after surgery.
  If the resected area exhibits signs of recurrence (e.g., wound nodulation, development of protuberances, pain, and/or itch), corticosteroid tape/plaster [17–19] can often extinguish the recurrence.
3. Lower jaw (Fig. 41)
  Pathological scars on the lower jaw often originate from acne and folliculitis.
  If the pathological scars lie among active acne lesions, the acne lesions should be the treatment priority.
  Small keloids and hypertrophic scars on the lower jaw can be treated with corticosteroid tape/plaster or injections but care should be taken to avoid aggravating any surrounding active acne lesions.
  The corticosteroid tape/plaster and injections can be combined with other conservative treatments such as oral medicines (e.g., tranilast).
  Medical therapies such as laser and make-up therapy can also be considered for pathological scars on the lower jaw.
  Surgery may also be a choice.
  If surgery is selected, it should be followed by postoperative radiotherapy or combined with corticosteroid tape/plaster or injection therapy [70–74, 76–78].
  The recommended postoperative radiotherapy is 15 Gy/3 fractions/3 days [77, 78].
  Surgery involves making an incision along the line of the lower jaw and then suturing it. The core excision method may be applied to remove the fibrous mass only.
  In terms of the sutures, it is recommended to use an absorbable thread that can maintain its tensile strength for a long time. For example, it is recommended to use 3-0 polydioxanone thread for the subcutaneous sutures and 4-0 or 5-0 polydioxanone thread for the dermal sutures.
  It is recommended to apply wound fixation materials for 3–6 months after surgery. The paper tape is an excellent choice because of its color and texture. However, paper tape can lead to contact dermatitis. In that case, the paper tape should be replaced with silicone tape until the contact dermatitis disappears.
  While fixation with silicone tape is also likely to be therapeutically effective, the currently available silicone tapes are slightly more noticeable after application than the paper tape.
  After surgery, a chin cap should be placed because this compression therapy may aid healing. However, it may be difficult to continue this treatment in summer. In that case, it is best to use fixation tape or gel sheeting alone [123, 124].
  If the resected area exhibits signs of recurrence (e.g., wound induration, bumps, pain, and/or itch), corticosteroid tape/plaster therapy should be started immediately.
4. Anterior chest wall (the scars developed from a midline chest incision) (Fig. 42)
  The first treatment choice for keloids and hypertrophic scars that develop after midline incision of the anterior chest is corticosteroid tape/plaster and injections.
  The corticosteroid tape/plaster and injection therapy may be accompanied by various conservative treatments such as oral medicines (e.g., tranilast).
  Medical treatments such as laser and make-up therapy can also be considered.
  If the scar is wide, surgery may be an option.
  If surgery is selected, it should be followed by postoperative radiotherapy [70, 72, 78].
  During surgery, the adipose tissue under the keloid/hypertrophic scar should also be resected. The deep fasciae of the right and left pectoris should then be firmly sutured with an absorbable thread. Thereafter, the superficial fascia should be firmly sutured. The deep and superficial fascial sutures should cause the wound edges to approximate each other closely. If that occurs, the dermal sutures can be started [18, 58, 61].
  In terms of the sutures, it is recommended to use an absorbable thread that can maintain its tensile strength for a long time. For example, it is recommended to use 0 or 2-0 polydioxanone thread for the deep fascial sutures, 3-0 polydioxanone thread for the subcutaneous sutures, and 4-0 or 5-0 polydioxanone thread for the dermal sutures.
  Z-plasty is not necessary if the incision is short and unlikely to be strongly stretched by body movements. However, if the incision is long and extends into the upper abdomen, a single z-plasty should be put on the infrasternal margin to decrease the tension on the wound.
  The recommended postoperative radiotherapy protocol is 20 Gy/4 fractions for 4 days [77, 78].
  Wound fixation should be performed for at least 6 months to 1 year after surgery. It is recommended to use silicone tape or gel sheets, which are resistant to contact dermatitis.
  For women, it is best to prevent horizontal tension by applying chest straps or bras.
  Radiation monotherapy may be considered for elderly people who do not meet the indications for surgery [77, 80].
5. Anterior chest wall (the scars developed from non-midline incisions or acne/folliculitis) (Figs. 43, 44, and 45)
  Most keloids and hypertrophic scars that spread laterally on the anterior chest arise from acne/folliculitis and minor surgery.
  Small scars that have strong hypertrophic scar properties (JSS 2015 score of 6–15 points [2]) can be treated with corticosteroid tape/plaster or injections.
  Large or multiple hypertrophic scar-like lesions can be treated with surgery. This is also true for lesions of any size that have strong keloid characteristics (JSS 2015 score of 16 or more points [2]).
  Conservative treatments on their own or in combination with similar therapies (e.g., oral medicines and laser and make-up therapies) may also be useful for anterior chest scars.
  If surgery is selected, it should be followed by postoperative radiotherapy [70, 72, 78]. However, radiotherapy should not be given to the areas that overlie the thyroid or mammary gland because these organs are at high risk of developing cancer. An exception to this rule may be elderly people because radiation sensitivity wanes with age.
  Surgery could be combined with therapies such as corticosteroid tape/plaster or injections, conservative treatments such as oral medicine, and medical treatments such as laser and make-up therapy.
  During surgery, the adipose tissue under the keloid/hypertrophic scar should also be resected. The deep fasciae of the pectoris major muscle should then be firmly sutured with an absorbable thread. Thereafter, the superficial fascia should be firmly sutured. The deep and superficial fascial sutures should cause the wound edges to approximate each other closely. If that occurs, the dermal sutures can be started [18, 58, 61].
  In terms of the sutures, it is recommended to use an absorbable thread that can maintain its tensile strength for a long time. For example, it is recommended to use 0 or 2-0 polydioxanone thread for the deep fascial sutures, 3-0 polydioxanone thread for the subcutaneous sutures, and 4-0 or 5-0 polydioxanone thread for the dermal sutures.
  Z-plasties should be applied if the postoperative wound is long: this will disrupt the horizontal tension that is placed on the wound by daily body movements.
  The recommended postoperative radiotherapy protocol is 20 Gy/4 fractions for 4 days [77, 78].
  Wound fixation materials should be applied for at least 6 months to 1 year after surgery. It is recommended to use silicone tape or gel sheets, which are resistant to contact dermatitis.
  For women, it is best to prevent horizontal tension by applying chest straps or bras.
  Radiation monotherapy may be considered for elderly people who do not meet the indications for surgery [77, 80].
6. Upper arm (Fig. 46)
  Most of the keloids and hypertrophic scars found on the upper arm develop from the BCG vaccination in childhood.
  Small pathological scars that have strong hypertrophic scar properties (JSS 2015 score of 6–15 points [2]) can be treated with corticosteroid tape/plaster or injections.
  Large or multiple hypertrophic scar-like lesions can be treated with surgery. This is also true for lesions of any size that have strong keloid characteristics (JSS 2015 score of 16 or more points [2]).
  If surgery is selected, it should be followed by postoperative radiotherapy [70, 72, 78].
  Surgery could be combined with therapies such as corticosteroid tape/plaster or injections, conservative treatments such as oral medicine, and medical treatments such as laser and make-up therapy.
  During surgery, the adipose tissue under the keloid/hypertrophic scar should also be resected, after which the subcutaneous tissue should be firmly sutured with an absorbable thread. It is recommended that the subcutaneous sutures are so firm that the wound edges naturally approximate each other. If so, the dermal sutures can be started [18, 58, 61].
  In terms of the sutures, it is recommended to use an absorbable thread that can maintain its tensile strength for a long time. For example, it is recommended to use 3-0 polydioxanone thread for the subcutaneous sutures and 4-0 or 5-0 polydioxanone thread for the dermal sutures.
  If the wound is long after resection, the tension on the wound can be released with some z-plasties.
  The recommended postoperative radiation protocol is 20 Gy/4 fractions for 4 days [77, 78].
  Wound fixation should be applied for at least 6 months to 1 year. It is recommended to use silicone tape or gel sheets, which are resistant to contact dermatitis. Applying a supporter or other compressive material may also aid wound healing.
  Radiation monotherapy may be considered for elderly people who do not meet the indications for surgery [77, 80].
7. Scapula (Figs. 47 and 48)
  Most of the keloids and hypertrophic scars that spread laterally on the scapular region are caused by acne/folliculitis and minor surgery.
  Small pathological scars that have strong hypertrophic scar properties (JSS 2015 score of 6–15 points [2]) can be treated with corticosteroid tape/plaster or injection therapy. Small keloid-like scars (JSS 2015 score of 16 or more points [2]) may also respond to this therapy.
  Large or multiple hypertrophic scar-like lesions can be treated with surgery. This is also true for lesions of any size that have strong keloid characteristics.
  If surgery is selected, it should be followed by postoperative radiotherapy [70, 72, 78].
  Surgery could be combined with therapies such as corticosteroid tape/plaster or injections, conservative treatments such as oral medicine, and medical treatments such as laser and make-up therapy.
  During surgery, the adipose tissue under the keloid/hypertrophic scar should also be resected, after which the subcutaneous tissue should be firmly sutured with an absorbable thread. It is recommended that the subcutaneous sutures are so firm that the wound edges naturally approximate each other. If so, the dermal sutures can be started [18, 58, 61].
  In terms of the sutures, it is recommended to use an absorbable thread that can maintain its tensile strength for a long time. For example, it is recommended to use 0 or 2-0 polydioxanone thread for the deep fascial sutures, 3-0 polydioxanone thread for the subcutaneous sutures, and 4-0 or 5-0 polydioxanone thread for the dermal sutures.
  If the post-resection wound is long, tension can be released with z-plasties.
  The recommended postoperative radiotherapy protocol is 20 Gy/4 fractions for 4 days [77, 78].
  Wound fixation should be performed for at least 6 months to 1 year. It is recommended to use silicone tape or gel sheets, which are resistant to contact dermatitis.
  Radiation monotherapy may be considered for elderly people who do not meet the indications for surgery [77, 80]
8. Joint areas (the hand, elbow, knee, and foot) (Fig. 49)
  Small pathological scars that have strong hypertrophic scar properties (JSS 2015 score of 6–15 points [2]) can be treated with corticosteroid tape/plaster or injections.
  Large or multiple hypertrophic scar-like lesions can be treated with surgery. This is also true for lesions of any size that have strong keloid characteristics (JSS 2015 score of 16 or more points [2]).
  If the scar has arisen from a surgical incision that runs in the direction in which the joint is extended, it is recommended to excise the whole lesion and apply z-plasties. This is especially true for narrow scars.
  In the case of wide lesions, it can be sufficient to simply divide the scars by introducing z-plasty and/or local flaps. This will effectively release the tension on the scar. Thus, total excision of the lesion is sometimes not necessary.
  When suturing the triangular flaps in z-plasty, it is important to use subcutaneous sutures to orient the flaps so that they can be easily transposed: the flaps should not be pulled into place by using dermal sutures.
  In terms of the sutures, it is recommended to use an absorbable thread that can maintain its tensile strength for a long time. For example, it is recommended to use 3-0 polydioxanone thread for the subcutaneous sutures and 4-0 or 5-0 polydioxanone thread for the dermal sutures.
  Surgery should be followed by either postoperative radiation or corticosteroid tape/plaster or injection therapy.
  Surgery can also be followed by oral medicines and laser and make-up therapies.
  The recommended postoperative radiation protocol is 15 Gy/3 fractions for 3 days [77, 78].
  The wound should be fixed after surgery with paper and silicone tapes or gel sheets. The joint should also be fixed with supporters or knee braces. Indeed, if thick materials such as gel sheets are used to fix the wound after surgery, it is advisable to apply pressure-fixation with supporters, knee braces, bandages, and similar materials. This is because the joint movements readily cause thick wound-fixation materials to fall off.
9. Abdomen (the scars developed from an abdominal midline incision) (Figs. 50 and 51)
  Surgery involving an abdominal midline incision often leads to keloid and hypertrophic scar development because the wounds are strongly stretched in the cephalocaudal direction due to the motion of the rectus abdominis muscle.
  Small pathological scars that have strong hypertrophic scar properties (JSS 2015 score of 6–15 points [2]) can be treated with corticosteroid tape/plaster or injections.
  Large or multiple hypertrophic scar-like lesions can be treated with surgery. This is also true for lesions of any size that have strong keloid characteristics (JSS 2015 score of 16 or more points [2]).
  Conservative treatments on their own or in combination with similar therapies (e.g., oral medicines and laser and make-up therapies) may also be useful for midline abdominal scars. However, the shape of the scar will remain after these therapies have achieved scar maturation.
  If surgery is selected, it will yield a thin linear scar that is almost invisible after maturation. Surgery should be followed by postoperative radiotherapy or corticosteroid tape/plaster and injection therapy.
  Surgery could be combined with conservative treatments such as oral medicine and medical treatments such as laser and make-up therapy.
  Indications for surgery include the age of the patient and, in the case of women, the desire to bear children.
  During surgery, the adipose tissue under the keloid/hypertrophic scar should also be resected. The deep fasciae of the underlying muscle should then be firmly sutured with an absorbable thread. Thereafter, the superficial fascia should be firmly sutured. The deep and superficial fascial sutures should cause the wound edges to approximate each other closely. If that occurs, the dermal sutures can be started [18, 58, 61].
  In terms of the sutures, it is recommended to use an absorbable thread that can maintain its tensile strength for a long time. For example, it is recommended to use 0 or 2-0 polydioxanone thread for the deep fascial sutures, 3-0 polydioxanone thread for the subcutaneous sutures, and 4-0 or 5-0 polydioxanone thread for the dermal sutures.
  When the post-resection wound is long, the tension may be released by applying z-plasties.
  The recommended postoperative radiotherapy protocol is 15 Gy in 3 fractions over 3 days [77, 78].
  Postoperative wound fixation should be performed with fixation tape or gel sheets for 6 months to 1 year.
  The abdomen should also be fixed by using abdominal bandages and a lumbosacral corset.
  Radiation monotherapy may be considered for elderly people who do not meet the indications for surgery [77, 80].
10. Abdomen (the scars developed from non-midline incisions) (Figs. 52 and 53)
  Another cause of pathological scars on the abdomen is abdominal transverse incision. The keloids and hypertrophic scars that arise from these incisions often have relatively mild symptoms.
  Endoscopic surgery can also lead to keloids and hypertrophic scars in the umbilical region and abdomen. In the umbilical region, infections often occur, in which case surgery is the first choice.
  Small pathological scars that have strong hypertrophic scar properties (JSS 2015 score of 6–15 points [2]) can be treated with corticosteroid tape/plaster or injection therapy.
  Large or multiple hypertrophic scar-like lesions can be treated with surgery. This is also true for lesions of any size that have strong keloid characteristics (JSS 2015 score of 16 or more points [2]).
  Conservative treatments on their own or in combination with similar therapies (e.g., oral medicines and laser and make-up therapies) may also be useful for non-midline abdominal scars.
  If surgery is selected, it should be followed with postoperative radiotherapy or corticosteroid tape/plaster or injection therapy.
  The indications for surgery include the age of the patient and, in the case of women, the desire to bear children.
  Non-midline abdominal scars can often be excised completely and then primarily sutured. This is because the abdomen has a relatively large amount of skin.
  During surgery, the adipose tissue just below the keloid/hypertrophic scar should also be resected. Thereafter, the deep fasciae of the underlying muscle should be firmly sutured with an absorbable thread. The superficial fascia should then be firmly sutured. The deep and superficial fascial sutures should cause the wound edges to approximate each other closely. If that occurs, the dermal sutures can be started [18, 58, 61].
  In terms of the sutures, it is recommended to use an absorbable thread that can maintain its tensile strength for a long time. For example, it is recommended to use 0 or 2-0 polydioxanone thread for the deep fascial sutures, 3-0 polydioxanone thread for the subcutaneous sutures, and 4-0 or 5-0 polydioxanone thread for the dermal sutures.
  When the post-resection wound is long, a single z-plasty performed in the middle of abdomen may release the tension in the horizontal direction.
  The recommended postoperative radiotherapy protocol is 15 Gy in 3 fractions over 3 days [77, 78].
  Postoperative wound fixation should be performed with fixation tape or gel sheets for 6 months to 1 year [125].
  The abdomen should also be fixed by using abdominal bandages and a lumbosacral corset.
  Radiation monotherapy may be considered for elderly people who do not meet the indications for surgery [77, 80].
11. Suprapubic (Fig. 54)
  The keloids and hypertrophic scars in the upper pubic region often originate from folliculitis.
  Small pathological scars that have strong hypertrophic scar properties (JSS 2015 score of 6–15 points [2]) can be treated with corticosteroid tape/plaster or injections.
  Large or multiple hypertrophic scar-like lesions can be treated with surgery. This is also true for lesions of any size that have strong keloid characteristics (JSS 2015 score of 16 or more points [2]).
  Surgery is also the first choice when pathological scars on the suprapubic region have an infection that is complicated by obstruction of the pores.
  If surgery is selected, it should be combined with postoperative radiotherapy or corticosteroid tape/plaster or injection treatment [70–74, 76–78].
  In terms of the sutures, it is recommended to use an absorbable thread that can maintain its tensile strength for a long time. For example, it is recommended to use 3-0 polydioxanone thread for the subcutaneous sutures and 4-0 or 5-0 polydioxanone thread for the dermal sutures.
  Surgery could also be combined with conservative treatments such as oral medicine.
  Hair removal by laser and other devices can also be considered.
  If the wound lengthens due to surgery, it is recommended to apply z-plasties to break the tension.
  The recommended postoperative radiotherapy protocol is 20 Gy/4 fractions/4 days [77, 78].
  Postoperative wound fixation should be performed with fixation tape or gel sheets for 6 months to 1 year [125].
  Radiation monotherapy may be considered for elderly people who do not meet the indications for surgery [77, 80].
12. Other body areas (Fig. 55)
  Although rare, keloids and hypertrophic scars can occur on the face, external genitals, and foot soles.
  Small pathological scars that have strong hypertrophic scar properties (JSS 2015 score of 6–15 points [2]) can be treated with corticosteroid tape/plaster or injections.
  Large or multiple hypertrophic scar-like lesions can be treated with surgery. This is also true for lesions of any size that have strong keloid characteristics (JSS 2015 score of 16 or more points [2]).
  Conservative treatments such as laser therapy may also be useful for these scars.
  If surgery is selected, it should be combined with postoperative radiotherapy or corticosteroid tape/plaster or injection therapy [70–74, 76–78].
  Surgery could also be combined with conservative treatments such as oral medicine.
  The postoperative radiotherapy protocol and the postoperative wound fixation method and duration should be selected on the basis of the body region and lesion severity.

## Conclusions

The JSW Consensus Document was published in Japan in 2018 and has been widely used throughout Japan ever since. It is likely that this Consensus Document will be constantly updated as basic research and practice progress. We would like to invite experts from around the world who are involved in the medical treatment of keloids and hypertrophic scars to contribute to future revisions, with the hope that we will become increasingly able to effectively improve the outcomes of patients who are susceptible to or are already suffering from keloids and hypertrophic scars.

## Data Availability

All data cited in this review can be found in their respective references.

## Abbreviations

5-FU: 5-Fluorouracil
BED: Biologically effective dose
BTX: Botulinum toxin
DFSP: Dermatofibrosarcoma protuberans
HLLT: High response level laser therapy
JSS: Japan Scar Workshop Scar Scale
JSW: Japan Scar Workshop
LLLT: Low response level laser therapy
NSAIDs: Non-steroidal anti-inflammatory drugs
ODT: Occlusive dressing technique
SCC: Squamous cell carcinoma
